# Isolation and identification of active ingredients and biological activity of *Dioscorea nipponica* Makino

**DOI:** 10.1186/s12906-023-04086-6

**Published:** 2023-07-17

**Authors:** Guangqing Xia, Guanshu Zhao, Shichun Pei, Yanping Zheng, Hao Zang, Li Li

**Affiliations:** 1 School of Pharmacy and Medicine, Tonghua Normal University, Tonghua, 134000 China; 2 Jilin Provincial Key Laboratory of Evaluation and Application of Changbai Mountain Biological Germplasm Resources, Tonghua, 134000 China; 3 Faculty of Engineering and IT, University of Technology Sydney, Sydney, 2007 Australia; 4 School of Food Science, Tonghua Normal University, Tonghua, 134000 China; 5 School of Chemistry, Tonghua Normal University, Tonghua, 134000 China

**Keywords:** *Dioscorea nipponica* Makino, Active ingredients, Separation and identification, Antioxidant activity, Inhibitory activity

## Abstract

This study reported the isolation and identification of bioactive compounds from *Dioscorea nipponica* Makino, a plant used in traditional medicine for various ailments. Nine compounds were isolated, including a new compound named as diosniposide E, which was elucidated by analyzing its ^1^H-NMR, ^13^C-NMR, DEPT, COSY, HMBC and MS data and comparing them with data available in literature. The other eight compounds were identified as known compounds. Theoretical calculations of energy and the generation of a molecular electrostatic potential surface map were employed to assess the antioxidant capacity of nine compounds, the calculation results exhibited that compounds **5** and **6** had strong antioxidant capacities. To further evaluate the antioxidant activities of the investigated compounds, the DPPH and ABTS assays were conducted. The results from the DPPH scavenging activity test revealed that compounds **4**–**6** exhibited enhanced scavenging activities compared to L-ascorbic acid, while displaying similar efficacy to trolox. Moreover, the ABTS scavenging activities of compounds **4**–**6** were found to surpass those of L-ascorbic acid and trolox. In terms of α-glucosidase inhibition, compounds **3** and **4** displayed remarkable inhibitory activities that surpassed the effects of acarbose. Additionally, compound **2** exhibited potent anticholinesterase activities, outperforming donepezil. This research provides insights into the potential bioactive compounds present in *Dioscorea* *nipponica* Makino and may contribute to its use in traditional medicine.

**Supplementary Information:**

The online version contains supplementary material available at 10.1186/s12906-023-04086-6.

## Introduction

Oxygen is a highly reactive element that can cause damage to biological molecules such as proteins and fats, resulting in various diseases such as cancer, aging, and diabetes. Free radicals generated by oxidative stress are responsible for the damage caused to biological molecules [1, 2]. Antioxidants can scavenge free radicals and protect against oxidative stress-induced damage [3]. Therefore, the identification of compounds with antioxidant and free radical scavenging activities is of great interest in the field of medicine and human health.

The decline in the body's capacity to counteract free radicals with age has emerged as the primary factor contributing to human aging and is implicated in 85% of diverse pathological conditions [4]. Scientific investigations have elucidated that reactive oxygen radicals can inflict harm upon vital biomolecules such as proteins and DNA through redox reactions, resulting in protein denaturation, cross-linking, enzyme activity impairment, and genetic mutations. Consequently, these events precipitate diminished immune function and heighten the susceptibility to cerebrovascular diseases, hepatic damage, and cancer [5–7]. Given this context, the supplementation of antioxidants has become an imperative strategy to combat the detrimental effects of these free radicals. Consequently, extensive scientific research has been directed towards identifying cost-effective and suitable antioxidants that can be readily assimilated by the human body. In this regard, natural antioxidants present a promising avenue for exploration and utilization, surpassing the prospects of synthetic antioxidants [8].

In the realm of anti-hypoxia research, natural drugs have garnered considerable attention due to their perceived lower incidence of toxic side effects when contrasted with chemical synthetic drugs. Extensive investigations have revealed the presence of anti-anoxia activity in both individual active constituents and combined formulations derived from botanical sources such as *Rhodiola rosea*, *Panax ginseng*, and *Panax notoginseng*, both in in vitro and in vivo experiments conducted on murine and rodent models [9, 10].

The genus *Dioscorea* encompasses a diverse array of plant species known for their rich reservoir of phytochemical constituents. These include steroidal saponins, flavonoids, diterpenoids, diphenylethane(ene)s, phenanthrenes, diarylheptanoids, phenylpropanoids, quinones, nitrogen-containing compounds, and organic acids. Remarkably, these phytochemicals are associated with a wide spectrum of biological activities, underscoring the versatile nature of *Dioscorea* plants. Notably, these plants exhibit substantial antioxidant, anti-inflammatory, hypoglycemic, hypolipidemic, antimicrobial, and antiproliferative properties [11]. *Dioscorea nipponica* Makino (*D. nipponica*), a prominent Chinese herb indigenous to the northern regions of China, possesses notable medicinal value primarily derived from its desiccated rhizome components [12]. Extensive prior investigations have highlighted its multifaceted therapeutic properties, including but not limited to lipid-lowering and anti-platelet aggregation effects, safeguarding hypoxic/reoxygenated cardiomyocytes, anti-aging attributes, and anti-tumor activities. Furthermore, *D. nipponica* is commonly employed in the treatment of Kashin-beck disease as well as muscle and bone numbness. Of particular interest, the steroidal saponins present in *D. nipponica* hold significance in synthesizing steroidal hormone drugs and are utilized in the management of cardiovascular conditions [13, 14].

*D. nipponica* is renowned for its folk medicinal usage in treating bronchitis and rheumatoid arthritis, remains relatively unexplored in terms of its antioxidant potential. This study aims to bridge this knowledge gap by presenting a comprehensive theoretical analysis focusing on the energetic aspects of deoxidation and oxidation, as well as the molecular electrostatic potential (MEP) surface map of *D. nipponica* constituents. Through a meticulous examination, we provide detailed mechanistic insights into the interactions involved and present a thorough comparative investigation of antioxidant activities. Simultaneously, in vitro antioxidant assays facilitated the isolation and purification of specific compounds from *D. nipponica* using silica-gel column chromatography, C_18_ column chromatography, and semi-preparative HPLC techniques. Structural elucidation of the isolated compounds was achieved through comprehensive interpretation of spectroscopic data, including ^1^H-NMR, ^13^C-NMR, DEPT, COSY, HMBC, and MS analysis, coupled with comparative analysis against existing literature reports. The antioxidant potential of these compounds was further investigated through theoretical comparative studies and validated through in vitro testing. Additionally, the inhibitory activities of these compounds against three enzymes were determined. The obtained results contribute to an enhanced understanding of the biological activity of *D. nipponica*.

## Material and methods

### Materials

The plant material (voucher specimen number: 2019-09-20-002) was collected from the wild in Tonghua (Jilin Province, China), and identified as the dried rhizome of *D. nipponica* by Prof. Jiamei Qin of Tonghua Normal University, and deposited at the Herbarium of Tonghua Normal University (Tonghua, China). We have permission to collect *D. nipponica* under the authorization of National Administration of Traditional Chinese Medicine.

### Methods

#### *D. nipponica* natural chemical composition extraction

The removal of soil, phellem and fibrous root system from the rhizomes of *D. nipponica* is an important step in preparing the plant material for further analysis. Collected samples were dried in a cool ventilated dry place, pulverized to powder and passed through a sieve (100 mesh). A total of 100 g of rhizome powder was subjected to degreasing using anhydrous diethyl ether in a Soxhlet extractor, employing reflux conditions for a duration of 2 h. The resulting material was subsequently dried. Following this, the obtained sample was subjected to extraction using 3000 mL of 60% ethanol at 60 °C for 2 h, 1.5 h and 1 h respectively, then the mixture was allowed to cool, filtered, and the ethanol extract was collected. 400 mL distilled water was added to the ethanol extract, and then extracted three times with equal volume of ethyl acetate, the combined ethyl acetate layer was then subjected to vacuum concentration until complete dryness was achieved.

#### Isolation of antioxidant compounds of *D. nipponica*

The use of ODS silica columns for liquid chromatography is common in the analysis of plant extracts. In this case, the ethyl acetate layer was dissolved in methanol and passed through the ODS silica column using gradient elution with methanol and water. The elution procedure determines the flow rate of the mobile phase (methanol and water) through the column and the proportion of each solvent used at various stages of the elution process. The elution procedure used for this study is described in Table 1. By using a gradient elution process, different eluates containing various compounds can be obtained and subsequently evaporated and dried by rotary evaporation, yielding separate samples for further analysis.

**Table 1:** Medium pressure liquid chromatography separation conditions

Time/min	Flow velocity/(mL·min^−1^)	A(Methanol)/%	B(Water)/%
0	10	5	95
10	10	20	80
20	10	40	60
40	10	70	30
50	10	90	10
60	10	100	0

#### Structure characterization of antioxidant compounds of *D. nipponica*

The obtained compounds were structurally characterized by electrospray mass spectrometry (ESI–MS) and nuclear magnetic resonance spectroscopy (NMR) analysis. The ionization parameters of ESI–MS: scanning range of m/z 100–1000, capillary voltage + 3.5 kV, capillary temperature 350 °C, sheath gas flow of 20 arbitrary units, and aux gas flow of 10 arbitrary units. NMR analysis was performed on a Varian NMR system 500 MHz, and the samples were dissolved in CD_3_OD solution, and the chemical shifts (δ values) of ^1^H and ^13^C spectra were obtained with TMS (tetramethylsilane) as the internal standard.

#### Evaluation of antioxidant activity of *D. nipponica*

##### Antioxidant activity evaluation using the DPPH assay

DPPH scavenging activity was conducted according to the literature [15]. Each sample (100 µL) in methanol at different concentrations (from 5 to 25 µM) was added to 100 µL of DPPH in methanol solution (50 µM). The solution was vortexed in 96-well plates for 10 s and then allowed to stand at room temperature for 20 min in the dark. The absorbance was measured at 492 nm on a microplate spectrophotometer. L-ascorbic acid and trolox were used as positive references. IC_50_ values (the concentrations required to scavenge 50% of the DPPH radicals present in the test solution) were calculated and expressed as the mean ± SD.

##### Antioxidant activity evaluation using the ABTS assay

ABTS scavenging activity was conducted according to the literature [16]. 1 mL of 2.6 mM potassium persulfate was added to 1 mL of 7 mM ABTS solution, and the mixture was incubated in the dark at room temperature for 12–16 h before use. The ABTS solution was diluted with methanol to provide an absorbance of 0.70 ± 0.02 at 734 nm. The diluted ABTS solution (190 µL) was added to sample fractions (10 µL) in DMSO at different concentrations. The plates were incubated at room temperature for 20 min in the dark. The absorbance was measured at 734 nm on a microplate spectrophotometer. L-Ascorbic acid and trolox were used as positive controls. The scavenging rate was expressed as % scavenging and was calculated as follows: IC_50_ values were calculated and expressed as the mean ± SD.

$$\%scavenging=\left(1-\frac{A_{sample}-A_{blank}}{A_{control}}\right)\times100\%$$

#### Evaluation of enzyme inhibitory activity of *D. nipponica*

##### Evaluation of cholinesterase inhibitory activity

Inhibitory activity against acetylcholinesterase (AChE) was assayed according to the literature [17]. Each sample in 10% DMSO solution (20 µL) was added to 120 µL phosphate buffer (pH 8.0, 0.1 M) and 20 µL AChE solution (pH 8.0, 0.8 U/mL, 0.1 M phosphate buffer). The mixture was incubated at 25 °C for 15 min. Then, 20 µL of iodothioacetylcholine (ATCI) solution (pH 8.0, 1.78 mM, in 0.1 M phosphate buffer) and 20 µL of 5,5'-dithiobis-(2-nitrobenzoic acid) (DTNB) solution (pH 8.0, 1.25 mM, in 0.1 M phosphate buffer) were added to each well, the mixture was incubated at 25 °C for 5 min. Before and after incubation, the absorbance was recorded at 405 nm on a microplate spectrophotometer. Donepezil was used as a positive control. The AChE inhibitory activity was expressed as % inhibition and was calculated as follows:

$$\%inhibition=\left(1-\frac{\triangle A_{sample}}{\triangle A_{control}}\right)\times100\%$$

Inhibitory activity against butyrylcholinesterase (BChE) was assayed according to the literature [17]. Each sample in 10% DMSO solution (20 µL) was added to 120 µL phosphate buffer (pH 8.0, 0.1 M) and 20 µL BChE solution (pH 8.0, 0.8 U/mL, in 0.1 M phosphate buffer). The mixture was incubated at 25 °C for 15 min. Then, 20 µL of butyrylthiocholine chloride solution (pH 8.0, 0.4 mM, 0.1 M phosphate buffer) and 20 µL of DTNB solution (pH 8.0, 1.25 mM, in 0.1 M phosphate buffer) were added to each well, the mixture was incubated at 25 °C for 5 min. Before and after incubation, the absorbance was recorded at 405 nm on a microplate spectrophotometer. Donepezil was used as a positive control. The BChE inhibitory activity was expressed as % inhibition and calculated as follows:

$$\%inhibition=\left(1-\frac{\triangle A_{sample}}{\triangle A_{control}}\right)\times100\%$$

##### Evaluation of α-glucosidase inhibitory activity

The α-glucosidase inhibitory activity was measured according to the literature [18]. Each sample (20 µL) in DMSO solution was added to 100 µL α-glucosidase solution (pH 6.9, 0.1 U/mL, in 0.1 M phosphate buffer). The mixture was vortexed in 96-well plates, incubated at 25 °C for 10 min. Then, 50 µL of p-nitrophenyl-α-D-glucopyranoside (pNPG) solution (pH 6.9, 5 mM, in 0.1 M phosphate buffer) was added to each well, the mixture was incubated at 25 °C for 5 min. Before and after incubation, the absorbance was recorded at 405 nm on a microplate spectrophotometer. Acarbose was used as a positive control. The α-glucosidase inhibitory activity was expressed as % inhibition and calculated as follows:

$$\%inhibition=\left(1-\frac{\triangle A_{sample}}{\triangle A_{control}}\right)\times100\%$$

All the experiments were carried out in triplicate and the data were analyzed using SPSS software (Version 22.0) and Origin software (Version 8.0).

#### Initial configuration

**Fig. 1:**
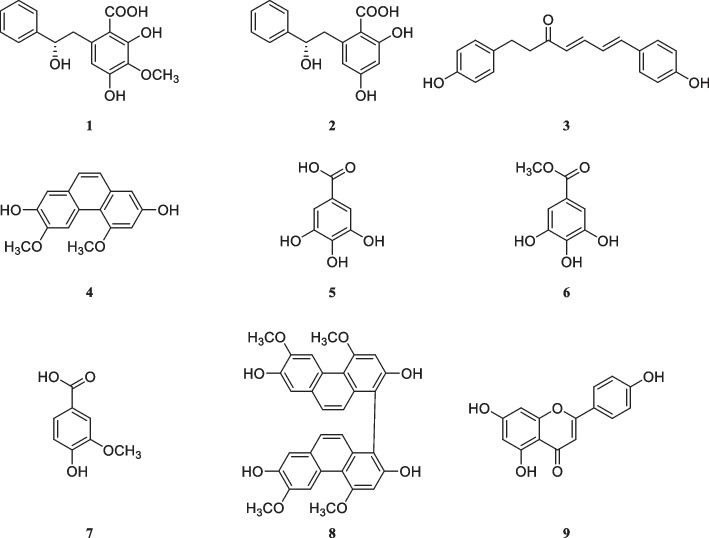
The initial trial configuration of compounds **1**–**9**

Phenolic compounds are commonly known for their antioxidant properties. Compounds **1**–**9** referred to in the study are phenolic compounds, they were selected for further investigation and their initial trial configurations are shown in Fig. 1.

#### Computational details

All molecular structures of the compounds were optimized utilizing the Gaussian16 program package at the B3LYP-D3/6-311G (d,p) level of theory [19–23]. The vibrational frequency calculations were conducted to confirm the structure at local minima. Furthermore, the open-shell energy calculations were performed to determine the redox reaction strength of the compounds at the UB3LYP-D3/6-311G (d,p) level. The Gaussview software and the VMD program were also used to produce MEP surface map of the molecules [21–23]. The energy of deoxidation (E_Deoxidation_) and oxidation (E_Oxidation_) were defined using the following formula to describe the redox reaction strength:

$$\begin{array}{c}{\mathrm E}_{\mathrm{Deoxidation}}={\mathrm E}_{\mathrm{Neutral}}-{\mathrm E}_{\mathrm e-}\\{\mathrm E}_{\mathrm{Oxidation}}={\mathrm E}_{\mathrm{Neutral}}-{\mathrm E}_{\mathrm e+}\end{array}$$

E_Deoxidation_ and E_Oxidation_ represent the energies of deoxidation and oxidation, respectively. Meanwhile, E_Neutral_, E_e −_ and E_e +_ refer to the energies of the corresponding system with neutral, e − and e + charge states. By adopting this definition, a smaller energy difference indicates a greater favorability for the reaction.

Collectively, the computational methods used in this study provide important insights into the electronic properties and chemical reactivity of the compounds, which are relevant to assessing their potential antioxidant activities.

## Results and discussion

### Structural identification of antioxidant compounds and theoretical model of *D. nipponica*

Nine phenolic compounds were isolated from the ethyl acetate layer of *D. nipponica* using medium pressure liquid chromatography and their structures were characterized clearly.

Compound **2**: Molecular Formula: C_15_H_14_O_5_, Molecular Weight:274. Yellow solid, ESI–MS m/z: 275.0546 [M + H]^+^. The ^1^H-NMR (500 MHz, CD_3_OD) spectrum of compound **2** showed the presence of seven aromatic protons at δ_H_ 7.52 (2H, m, H-2,6), 7.44 (2H, m, H-3,5), 7.39 (1H, m, H-4), 6.28 (1H, s, H-10), 6.25 (1H, s, H-12), an oxygenated methine proton at 5.61 (1H, dd, J = 11.5, 3.0, H-7), a methylene proton at 3.25 (1H, m, H-8a) and 3.11 (1H, dd, J = 16.5, 3.5, H-8b); ^13^C-NMR (125 MHz) δ 170.1 (14-COOH), 165.0 (C-11), 164.3 (C-13), 138.7 (C-1), 142.0 (C-9), 128.3 (C-3,4,5), 125.9 (C-2,6), 106.6 (C-10), 100.9 (C-12), 100.3 (C-14), 80.5 (C-7), 34.7 (C-8). The compound was identified as diosniponol C, by comparison of data to that of known compounds in literature [24]. The ^13^C NMR spectrum (Table 2) displayed the appearance of 15 carbon signals, including a carbonyl carbon at δ_C_ 170.1, one oxygenated methine carbon at δC 80.5, one methylene carbon δ_C_ 34.7, and 12 aromatic carbons.

**Table 2:** ^1^H and ^13^C NMR data of compound **1** and **2** in CD_3_OD

Position	**1**			**2**		
	*δ*_H_ (J, H_Z_)		*δ*_C_	*δ*_H_ (J, H_Z_)		*δ*_C_
1	—	138.6		—	138.7	
2	7.52 m	125.9		7.52 m	125.9	
3	7.44 m	128.3		7.44 m	128.3	
4	7.39 m	128.3		7.39 m	128.3	
5	7.44 m	128.3		7.44 m	128.3	
6	7.52 m	125.9		7.52 m	125.9	
7	5.61 dd (11.5, 3.0)	80.8		5.61 dd (11.5, 3.0)	80.5	
8	3.25 m	34.2		3.25 m	34.7	
	3.11 dd (16.5, 3.5)			3.11 dd (16.5, 3.5)		
9	—	136.0		—	142.0	
10	6.37 s	106.5		6.28 s	106.6	
11	—	157.2		—	165.0	
12	—	133.8		6.25 s	100.9	
13	—	156.3		—	164.3	
14	—	101.0		—	100.3	
14-COOH	—	170.3		—	170.1	
12-OCH_3_	3.86 s	59.5				

Compound **1**, molecular formula: C_16_H_16_O_6_, molecular weight: 304. Yellow solid, mp 166.0–168.0 °C; HR-ESI–MS m/z: 305.0644 [M + H]^+^ (theoretical value 304.0647), supporting the presumed molecular formula C_16_H_16_O_6_; The ^1^H-NMR (500 MHz, CD_3_OD) spectrum of compound **1** showed the presence of six aromatic protons at δ_H_ 7.52 (2H, m, H-2,6), 7.44 (2H, m, H-3,5), 7.39 (1H, m, H-4), 6.37 (1H, s, H-10). an oxygenated methine proton at 5.61 (1H, dd, J = 11.5, 3.0, H-7), 3.86 (3H, s, 12-OCH_3_), a methylene proton at 3.25 (1H, m, H-8a) and 3.11 (1H, dd, J = 16.5, 3.5, H-8b); ^13^C-NMR (125 MHz) δ 170.3 (14-COOH), 157.2 (C-11), 156.3 (C-13), 138.6 (C-1), 136.0 (C-9), 128.3 (C-3,4,5), 125.9 (C-2,6), 106.5 (C-10), 133.8 (C-12), 101.0 (C-14), 80.8 (C-7), 59.5 (12-OCH_3_), 34.2 (C-8). The ^13^C NMR spectrum (Table 2) displayed the appearance of 16 carbon signals, including a carbonyl carbon at δ_C_ 170.3, one oxygenated methine carbon at δ_C_ 80.8, one methylene carbon δ_C_ 34.2, and 12 aromatic carbons. DEPT-90 and 135 displayed the existence of the following functional groups: one **-**CH_3_, one **-**CH_2_, seven **-**CH, seven **-**C, and one **-**C = O.

**Fig. 2:**
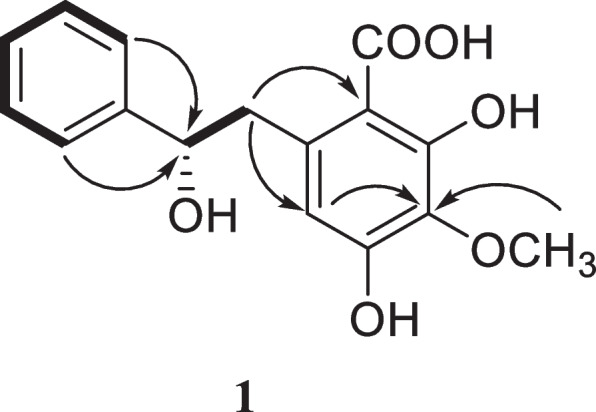
^1^H–^1^H COSY (boldlines) and HMBC (arrow) correlations for compound **1**

A strong correlation between H-7 and H-8, as well as between H-2,6 and H-3,5 can be clearly seen in the COSY spectrum, indicating that these hydrogens are connected. Furthermore, the HMBC spectrum confirms correlations between C-7 and H-2, C-7 and H-6, C-10 and H-8, C-14 and H-8, C-12 and H-10, C-12 and 12-OCH_3_. The connection of one methoxy group was confirmed to be at C-12 by HMBC cross-peaks of 12-OCH_3_/C-12. (Fig. 2). A comparison with the known compound **2,** it can be seen that the absence of a hydrogen on the benzene ring and the presence of an additional methoxy group in ^1^H-NMR spectrum of the new compound **1**; the optical rotation of compound **1** exhibited a positive value ([α]^25^D + 19.0° MeOH), and the absolute configuration of the new compound C7 in comparison with known literature is in the S form [24], therefore it was inferred that compound **1** was named as diosniposide E.

Compound **3**: Molecular Formula: C_19_H_18_O_3_, Molecular Weight: 294. Yellow solid, EI-MS m/z: 294. ^1^H-NMR (500 MHz, CD_3_OD) δ 7.35 (2H, d, J = 8.5 H-2′′,6′′), 7.28 (1H, dd, J = 15.5, 10.8, H-5), 6.98 (2H, d, J = 8.5, H-2′,6′), 6.95 (1H, d, J = 15.5, H-7), 6.80 (1H, dd, J = 15.5, 10.8, H-6), 6.63 (2H, d, J = 8.5, H-3′,5′), 6.62 (2H, J = 8.5, H-3′′,5′′), 6.18 (1H, d, J = 15.5, H-4), 2.82 (2H, t, J = 7.4, H-1), 2.73 (2H, t, J = 7.4, H-2). ^13^C-NMR (125 MHz) δ 198.7 (C-3), 158.7 (C-4′′), 155.4 (C-4′), 143.3 (C-5), 141.4 (C-7), 131.1 (C-1′), 128.9 (C-2′,6′), 128.8 (C-2′′,6′′), 127.8 (C-4), 127.0 (C-1′′), 123.6 (C-6), 115.6 (C-3′′,5′′), 41.6 (C-2), 29.0 (C-1). The data are consistent with the literature [25] and the compound was identified as 1,7-bis(4-hydroxyphenyl)hepta-4E,6E-dien-3-one.

Compound **4**: Molecular Formula: C_16_H_14_O_4_, Molecular Weight: 270. Yellow solid, EI-MS m/z: 270. ^1^H-NMR (500 MHz, CD_3_OD) δ 7.79 (1H, s, H-7), 7.36, 7.27 (2H, d, J = 8.7, H-12,13), 7.06 (1H, s, H-10), 6.70 (1H, d, J = 2.5, H-1), 6.63 (1H, d, J = 2.5, H-3), 3.99 (3H, s, 8-OCH_3_), 3.93 (3H, s, 4-OCH_3_); ^13^C-NMR (125 MHz) δ 159.2 (C-2), 154.8 (C-4), 147.5 (C-8), 144.5 (C-9), 134.8 (C-14), 127.2 (C-11), 126.6 (C-13), 124.6 (C-12), 124.3 (C-6), 114.5 (C-5), 111.4 (C-10), 108.6 (C-1), 104.2 (C-7), 98.6 (C-3), 54.8 (8-OCH_3_), 54.7 (4-OCH_3_). The data were consistent with the literature [26] and the compound was identified as 3,5-dimethoxyphenanthrene-2,7-diol.

Compound **5**: Molecular Formula: C_7_H_6_O_5_, Molecular Weight: 170. Yellow solid, EI-MS m/z: 170. ^1^H-NMR (500 MHz, Acetone-d_6_) δ 7.06 (2H, s, H-2,6); ^13^C-NMR (125 MHz) δ 170.8 (C-7), 146.9 (C-3,5), 139.6 (C-4), 122.4 (C-1), 110.5 (C-2,6). The data are consistent with the literature [27] and the compound was identified as gallic acid.

Compound **6**: Molecular Formula: C_8_H_8_O_5_, Molecular Weight: 184. Yellow solid, EI-MS m/z: 184. ^1^H-NMR (500 MHz, Acetone-d_6_) δ 7.11 (2H, H-2,6), 3.78 (3H, s, 7-OCH_3_). ^13^C-NMR (125 MHz) δ 167.3 (C-7), 146.1 (C-3,5), 138.8 (C-4), 121.9 (C-1), 109.9 (C-2,6), 52.0 (7-OCH_3_). The data were consistent with the literature [27] and the compound was identified as methyl gallate.

Compound **7**: Molecular Formula: C_8_H_8_O_4_, Molecular Weight: 168. Yellow solid, EI-MS m/z: 168. ^1^H-NMR (500 MHz, Acetone-d_6_) δ 7.59 (1H, m, H-6), 7.55 (1H, m, H-2), 6.90 (1H, d, J = 2.5, H-5), 3.90 (3H, s, 3-OCH_3_); ^13^C-NMR (125 MHz) 167.7 (C-7), 152.2 (C-4), 148.2 (C-3), 124.9 (C-6), 123.0 (C-1), 115.6 (C-2), 113.5 (C-5), 56.4 (3-OCH_3_). The data were consistent with the literature [28] and the compound was identified as vanillic acid.

Compound **8**: Molecular Formula: C_32_H_26_O_8_, Molecular Weight: 538. Yellow solid, EI-MS m/z: 538. ^1^H-NMR (500 MHz, Acetone-d_6_) δ 9.24 (2H, s, H-5,5′), 7.28 (2H, d, J = 9.2, H-9,9′), 7.12 (2H, s, H-8,8′), 7.00 (2H, s, H-3,3′), 6.91 (2H, d, J = 9.2, H-10,10′), 4.23 (6H, s, 4,4′-OCH_3_), 4.09 (6H, s, 6,6′-OCH_3_); ^13^C-NMR (125 MHz) δ 166.4 (C-2,2′), 158.1 (C-4,4′), 147.7 (C-6,6′), 145.0 (C-7,7′), 133.8 (C-10a,10a′), 126.3 (C-8a,8a′,9,9′), 124.1 (C-4b,4b′), 122.5 (C-10,10′), 115.2 (C-1,1′), 114.1 (C-4a,4a′), 111.6 (C-8,8′), 109.1 (C-5,5′), 99.0 (C-3,3′), 55.1 (4,4′,6,6′-OCH_3_). The data were consistent with the literature [29] and the compound was identified as 2,2',7,7'-tetrahydroxy-4,4',6,6'-tetramethoxy-1,1'-biphenanthrenes.

**Fig. 3:**
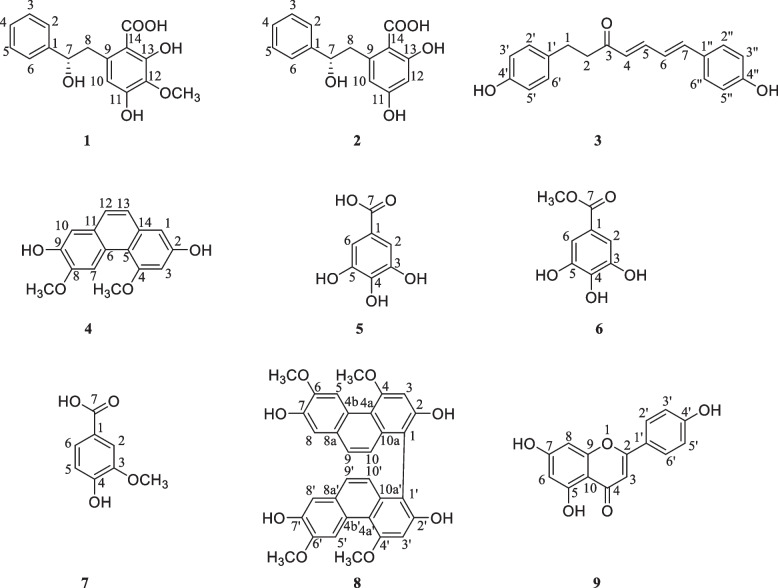
Chemical structures of compounds **1**–**9**

Compound **9**: Molecular Formula: C_15_H_10_O_5_, Molecular Weight: 270. Yellow solid, EI-MS m/z: 270. ^1^H-NMR (500 MHz, Acetone-d_6_) δ 12.95 (1H, s, 5-OH), 7.91 (2H, d, J = 8.8, H-2',6'), 6.90 (2H, d, J = 8.8, H-3',5'), 6.75 (1H, s, H-3), 6.47 (1H, d, J = 2.0, H-8), 6.18 (1H, d, J = 2.0, H-6); ^13^C NMR (125 MHz) 181.8 (C-4), 164.3 (C-7), 163.8 (C-2), 161.5 (C-5), 161.2 (C-4'), 157.4 (C-9), 128.5 (C-2',6'), 121.2 (C-1'), 116.0 (C-3',5'), 103.7 (C-10), 102.8 (C-3), 98.9 (C-6), 94.0 (C-8). The data were consistent with the literature [30] and the compound was identified as apigenin. The chemical structures of the nine compounds are shown in Fig. 3.

#### Theoretical models

**Fig. 4:**
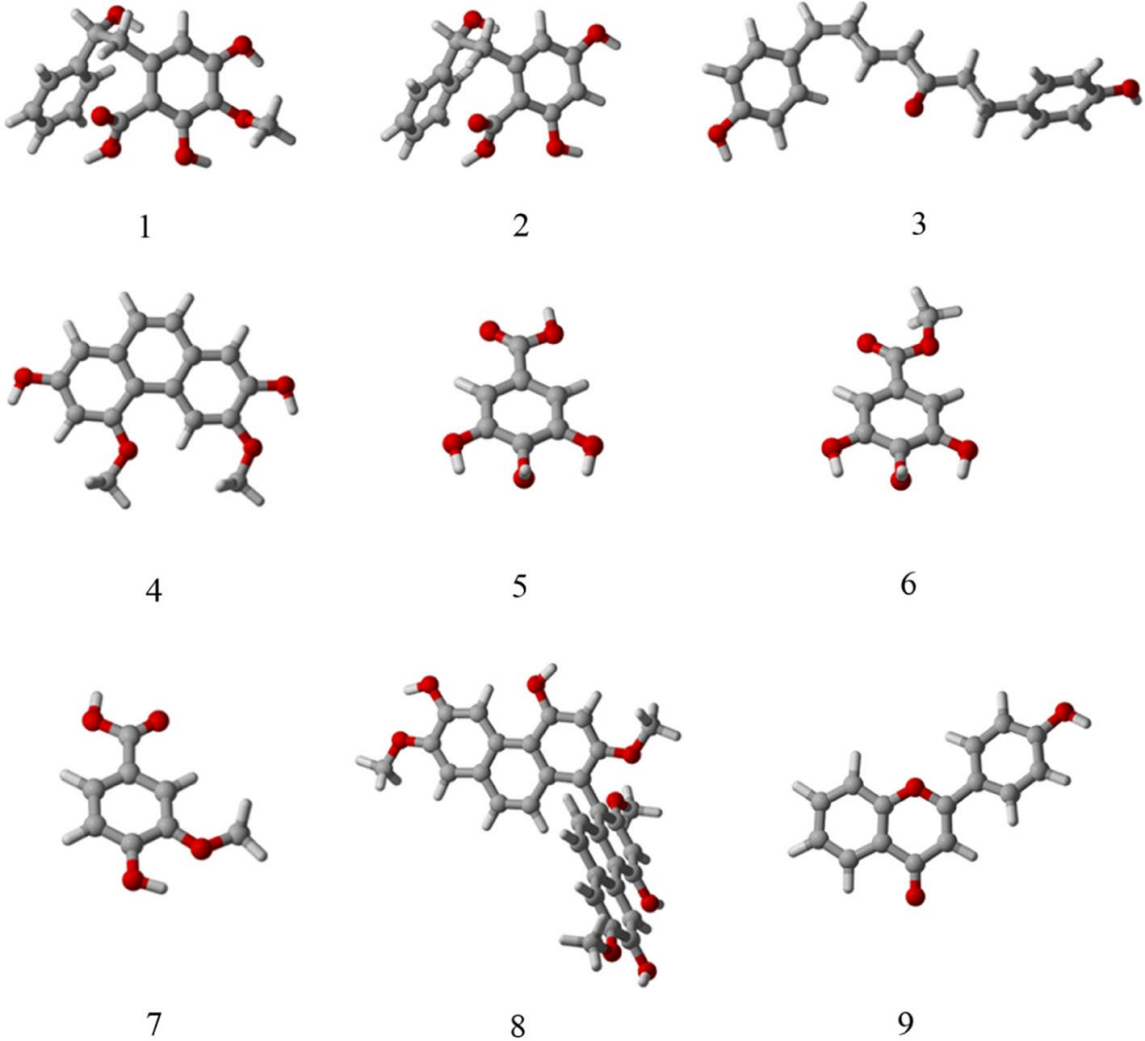
Several optimized configurations of compounds **1**–**9**

Figure 4 displays multiple optimized configurations for compounds **1–9**. Through density functional theory simulations, it has been determined that these theoretical models align well with the initial trial configurations depicted in Fig. 1. This consistency further validates the accuracy and reliability of the theoretical calculations performed in this study.

#### Energy

The deoxidation and oxidation energies of compounds **1**–**9** have been calculated to assess the strength of their redox reactions. A smaller energy difference between deoxidation and oxidation signifies a favorable reaction. Table 3 presents the results, indicating that the energies of deoxidation are lower than those of oxidation for all compounds. This suggests that compounds **1**–**9** are more inclined towards reduction rather than oxidation. Notably, compounds **5**–**7** exhibit significantly stronger abilities to acquire electrons compared to losing electrons, suggesting their potential for enhanced antioxidant capacity. These findings imply that compounds **5**–**7** may possess stronger antioxidant properties due to their pronounced electron-acquiring abilities.

**Table 3:** The deoxidation and oxidation energies of compounds **1**–**9**

Compound	Energy (eV)				
	E _Deoxidation_	E _e−_	E _Neutral_	E _e+_	E _Oxidation_
1	− 0.11	− 1071.00	− 1071.00	− 1070.73	− 7.56
2	− 0.17	− 956.44	− 956.45	− 956.16	− 7.79
3	+ 0.98	− 960.83	− 960.79	− 960.53	− 7.03
4	− 0.34	− 919.27	− 919.29	− 919.04	− 6.55
5	− 0.07	− 646.68	− 646.68	− 646.38	− 8.13
6	− 0.12	− 685.99	− 685.99	− 685.70	− 8.02
7	− 0.33	− 610.74	− 610.76	− 610.47	− 7.87
8	− 0.06	− 1837.37	− 1837.37	− 1837.16	− 5.89
9	+ 0.60	− 803.54	− 803.52	− 803.23	− 7.71

#### MEP surface map

**Fig. 5:**
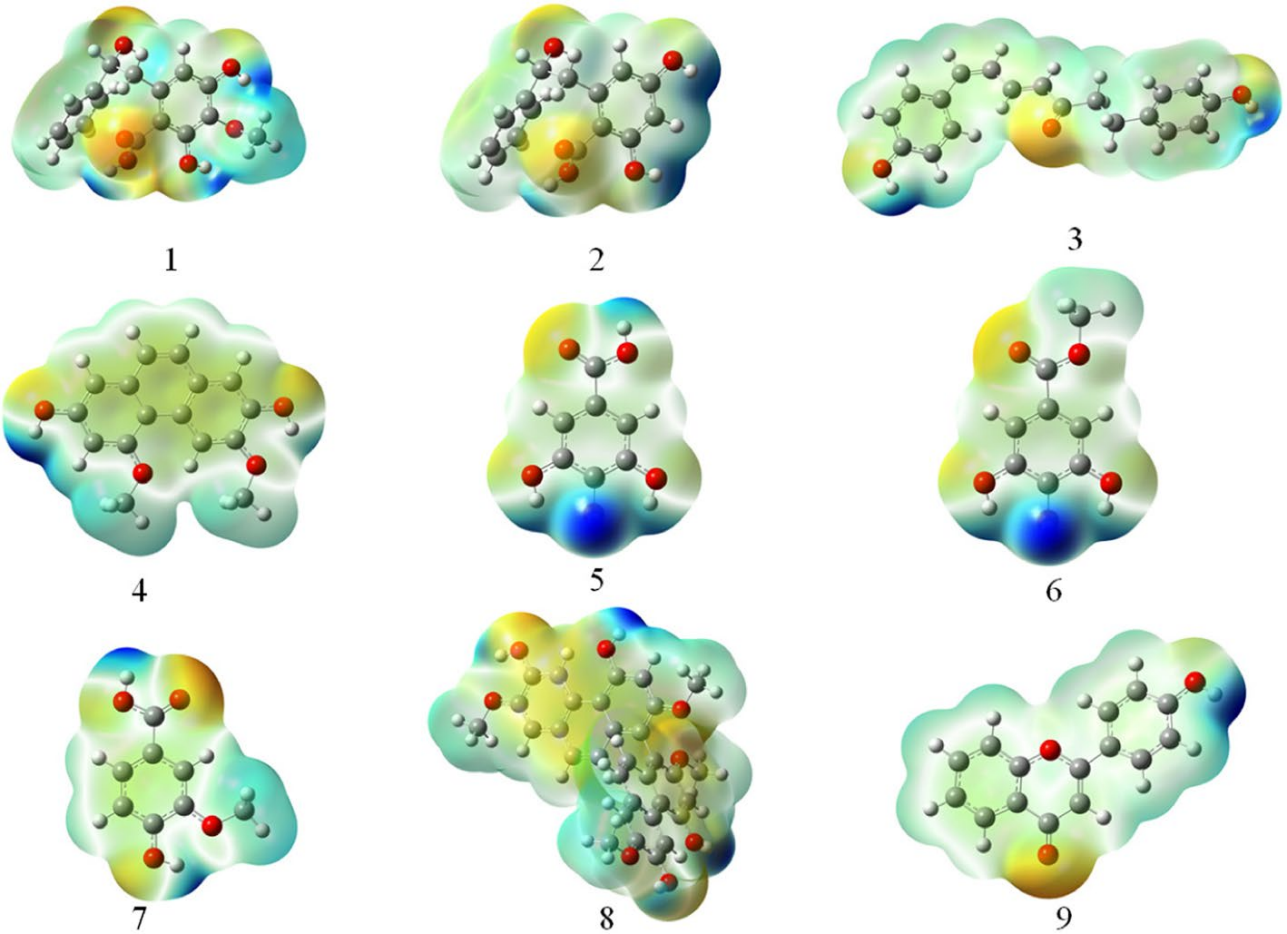
MEP surface map of compounds **1**–**9**

Figure 5 displays the MEP surface maps of nine compounds and offers a visual method to comprehend the relative polarity of each molecule. The different values of the MEP at the surface are depicted using different colors, with blue signifying the regions of the most positive electrostatic potential and red representing the regions of the most negative electrostatic potential. As the principle of an antioxidant is to lower the concentration of oxygen through reduction, the activated site for antioxidant activity is predicted to be the region of the positive electrostatic potential, with darker areas of blue being preferred. As illustrated in Fig. 5, compounds **5** and **6** exhibited more positive electrostatic potential than the other compounds, which is consistent with the energy calculation results.

#### Analysis of the antioxidant activity of *D. nipponica*

Table 4 shows the free radical-scavenging activities of compounds **1**–**9** in comparison with L-ascorbic acid and trolox according to the DPPH assay. compounds **4**–**6** exhibited high scavenging activity, which is comparable to trolox and superior to L-ascorbic acid. In contrast, the remaining six compounds exhibit relatively weak scavenging activity in the assay. These findings emphasize the potential of compounds **4**–**6** as effective DPPH scavengers, suggesting their promising antioxidant capabilities.

**Table 4:** Scavenging effect of antioxidant compounds of *D. nipponica* on DPPH and ABTS

Compound	DPPH IC_50_ (μM)	ABTS IC_50_ (μM)
**1**	> 5000	27.31 ± 0.21
**2**	> 5000	26.97 ± 0.19
**3**	> 5000	26.62 ± 0.13
**4**	3.09 ± 0.08	3.41 ± 0.02
**5**	2.97 ± 0.07	3.26 ± 0.04
**6**	3.82 ± 0.10	4.19 ± 0.06
**7**	2509.12 ± 10.11	29.22 ± 0.28
**8**	> 5000	352.16 ± 0.07
**9**	> 5000	498.81 ± 5.32
L-Ascorbic acid	9.86 ± 0.33	24.84 ± 0.31
Trolox	2.50 ± 0.02	18.30 ± 0.22

Table 4 shows that compounds **4**–**6** exhibited strong scavenging activity at low concentrations, with their IC_50_ values being 6–7 times lower than that of the positive controls (L-ascorbic acid and trolox). In addition, compounds **1**–**3** and **7** also exhibited strong scavenging activity, similar to L-ascorbic acid. However, compounds **8** and **9** had weaker scavenging activity compared to the other compounds tested.

In the search for natural free radical scavengers, both DPPH and ABTS assays are considered to be fast, effective, and economical methods, due to their simplicity, low cost, and reproducibility. These methods can be used to evaluate the antioxidant activity of various compounds, and the results provide valuable information on the potential health benefits of these compounds. DPPH is a fat-soluble free radical and ABTS is a water-soluble free radical. The capacity to scavenge ABTS is considered a more representative measure of antioxidant performance within the human body, as it corresponds to the water-soluble environment. Based on this consideration, compounds **1–7** exhibit potential antioxidant properties. Consequently, these compounds warrant further research and development to explore their efficacy and potential applications as antioxidants.

Compound **4**, categorized as a phenanthrene derivative, aligns with previous reports on phenanthrene derivatives derived from *Dioscorea communis*, which have demonstrated notable scavenging activities against DPPH and ABTS [31]. The data from the literature mentioned above are consistent with our experimental results. Compound **5** is a naturally occurring substance found in various plants, vegetables, nuts and fruits [32]. It has been recognized as a potent antioxidant in emulsion or lipid systems, making it widely applicable in food packaging, preservation, and cosmetics [33]. Importantly, compound **5** has demonstrated non-toxicity when orally administered at a dose of 5000 mg/kg body weight [34]. Similarly, compound **6** has exhibited strong antioxidant activity without inducing any adverse effects even at a dosage of 1000 mg/kg [35]. Furthermore, compound **7** has been found to effectively reduce reactive oxygen species production and scavenge free radicals [36]. These observations highlight the potential of these three compounds as superior antioxidants compared to existing products in the market. Considering their natural origin, powerful antioxidant properties, and absence of significant adverse effects, further research on these compounds is warranted.

The antioxidant activities of compounds **1–4** have not been previously reported, making this study the first to assess their antioxidant potential. However, further comprehensive and systematic evaluations are necessary before these compounds can be established as antioxidants. In future research, when additional antioxidant compounds are isolated from the ethanol extract of *D. Nipponica*, the extract itself can be considered for antioxidant investigations. By expanding the scope of research and identifying more antioxidant compounds, a more comprehensive understanding of the antioxidant capacity of *D. Nipponica* and its extracts can be achieved.

#### Analysis of the enzyme inhibitory activity of *D. nipponica*

The results for the α-glucosidase inhibitory activities of compounds **1**–**9** as evaluated using the α-glucosidase inhibition assay in comparison with acarbose are summarized in Table 5. Compounds **3** and** 4** demonstrated significantly higher α-glucosidase inhibitory activities compared to acarbose, which is a recognized α-glucosidase inhibitor. These two compounds show promising potential as α-glucosidase inhibitors. However, the remaining seven compounds exhibited weaker α-glucosidase inhibitory activities when compared to acarbose. Further investigations and structure–activity relationship studies are warranted to understand the variations in α-glucosidase inhibitory activities among these compounds.

**Table 5:** Inhibitory activities of compounds **1**–**9** on α-glucosidase and cholinesterase

Compound	α-Glucosidase IC_50_ (μM)	AChE IC_50_ (μM)	BChE IC_50_ (μM)
**1**	> 800	113.10 ± 0.04	58.12 ± 0.12
**2**	> 800	0.08 ± 0.00	3.02 ± 0.12
**3**	1.60 ± 0.17	862.10 ± 5.85	321.69 ± 2.14
**4**	31.4 ± 0.18	425.20 ± 2.66	265.9 ± 1.79
**5**	> 800	> 1000	> 1000
**6**	> 800	> 1000	> 1000
**7**	> 800	> 1000	> 1000
**8**	> 800	> 1000	> 1000
**9**	> 800	> 1000	> 1000
Acarbose	60.87 ± 1.02	Not tested	Not tested
Donepezil	Not tested	0.10 ± 0.00	3.58 ± 0.08

Compound **2** demonstrated the highest inhibitory activity against both AChE and BChE (Table 5), Notably, the inhibitory activity of compound **2** surpassed that of donepezil, a well-known cholinesterase inhibitor. This finding suggests that compound **2** has the potential to be developed as a promising cholinesterase inhibitor. However, it is worth mentioning that the remaining eight compounds exhibited weaker cholinesterase inhibitory activities compared to donepezil. Further investigations and structure–activity relationship studies are needed to explore the underlying mechanisms.

## Conclusion

A total of nine compounds were successfully isolated from the ethyl acetate layer of the ethanol extract of *D. nipponica* rhizomes. Among these compounds, eight were previously known and have been identified (diosniponol C, 1,7-bis(4-hydroxyphenyl)hepta-4E,6E-dien-3-one, 3,5-dimethoxyphenanthrene-2,7-diol, gallic acid, methyl gallate, vanillic acid, 2,2',7,7'-tetrahydroxy-4,4',6,6'-tetramethoxy-1,1'-biphenanthrenes and apigenin) and one new compound (diosniposide E), which differed from the known compound diosniponol C, only by one methoxy substitution at C-12.

Theoretical calculations of energy and MEP surface maps were performed to assess the redox properties of compounds **1**–**9**. The outcomes of these calculations revealed that the investigated compounds have a higher tendency towards reduction, with compounds **5** and **6** exhibiting notably stronger electron acquisition abilities compared to electron loss. This observation suggests that compounds **5** and **6** may possess enhanced antioxidant activities. Subsequently, a comprehensive screening was conducted to evaluate the in vitro antioxidant activity and enzyme inhibitory potential of the nine compounds. Compounds **4**–**6** showed robust scavenging activities against DPPH and ABTS radicals, surpassing the efficacy of L-ascorbic acid and demonstrating similar potency to trolox in the DPPH assay, and surpassing both L-ascorbic acid and trolox in the ABTS assay. Furthermore, compounds **3** and **4** exhibited significant inhibitory effects on α-glucosidase, surpassing the activity of the standard acarbose. Notably, compound **2** displayed noteworthy inhibitory activity against both AChE and BChE, outperforming the reference compound donepezil. These findings lay a solid foundation for further research and development of natural antioxidants, antidiabetic agents and anticholinesterase drugs derived from *D. nipponica*.

## Supplementary Information

**Additional file 1: Figure S1**. ^1^HNMR spectrum of compound **1**. **Figure S2**. ^13^C NMR spectrum of compound **1**. **Figure S3**. DEPT spectrum of compound **1**. **Figure S4**. COSY spectrum of compound **1**. **Figure S5**. HMBC spectrum of compound **1**. **Figure S6**. HRESIMS spectrum of compound **1**.** Figure S7**. ^1^H NMR spectrum of compound **2**. **Figure S8**. ^13^C NMR spectrum of compound **2**. **Figure S9**. HRESIMS spectrum of compound **2**. **Figure S10**. ^1^HNMR spectrum of compound **3**. **Figure S11**. ^13^C NMR spectrum of compound **3**. **Figure S12**. EIMS spectrum of compound **3**. **Figure S13**. ^1^H NMR spectrum of compound **4**. **Figure S14**. ^13^C NMR spectrum of compound **4**. **Figure S15**. EIMS spectrum of compound **4**. **Figure S16**. ^1^H NMR spectrum of compound **5**. **Figure S17**. ^13^C NMR spectrum of compound **5**. **Figure S18**. ^1^H NMR spectrum of compound **6**. **Figure S19**. ^13^C NMR spectrum of compound **6**. **Figure S20**. EIMS spectrum of compound **6**. **Figure S21**. ^1^H NMR spectrum of compound **7**. **Figure S22**. ^13^C NMR spectrum of compound **7**. **Figure S23**. EIMS spectrum of compound **7**. **Figure S24**. ^1^H NMR spectrum of compound **8**. **Figure S25**. ^13^C NMR spectrum of compound **8**. **Figure S26**. EIMS spectrum of compound **8**. **Figure S27**. ^1^H NMR spectrum of compound **9**. **Figure S28**. ^13^C NMR spectrum of compound **9**. **Figure S29**. EIMS spectrum of compound **9**.

## Data Availability

The datasets used and/or analysed during the current study are available from the corresponding author on reasonable request.
