# Supplementary material for: Isolation and identification of active ingredients and biological activity of Dioscorea nipponica Makino
Source: BMC Complement Med Ther. 2023 Jul 17;23:240. doi: 10.1186/s12906-023-04086-6 (PMC10351190; doi:10.1186/s12906-023-04086-6)
Supplement: Supplementary file 1 — Additional file 1: Figure S1. 1HNMR spectrum of compound 1. Figure S2. 13C NMR spectrum of compound 1. Figure S3. DEPT spectrum of compound 1. Figure S4. COSY spectrum of compound 1. Figure S5. HMBC spectrum of compound 1. Figure S6. HRESIMS spectrum of compound 1. Figure S7. 1H NMR spectrum of compound 2. Figure S8. 13C NMR spectrum of compound 2. Figure S9. HRESIMS spectrum of compound 2. Figure S10. 1HNMR spectrum of compound 3. Figure S11. 13C NMR spectrum of compound 3. Figure S12. EIMS spectrum of compound 3. Figure S13. 1H NMR spectrum of compound 4. Figure S14. 13C NMR spectrum of compound 4. Figure S15. EIMS spectrum of compound 4. Figure S16. 1H NMR spectrum of compound 5. Figure S17. 13C NMR spectrum of compound 5. Figure S18. 1H NMR spectrum of compound 6. Figure S19. 13C NMR spectrum of compound 6. Figure S20. EIMS spectrum of compound 6. Figure S21. 1H NMR spectrum of compound 7. Figure S22. 13C NMR spectrum of compound 7. Figure S23. EIMS spectrum of compound 7. Figure S24. 1H NMR spectrum of compound 8. Figure S25. 13C NMR spectrum of compound 8. Figure S26. EIMS spectrum of compound 8. Figure S27. 1H NMR spectrum of compound 9. Figure S28. 13C NMR spectrum of compound 9. Figure S29. EIMS spectrum of compound 9. [file 12906_2023_4086_MOESM1_ESM.doc]

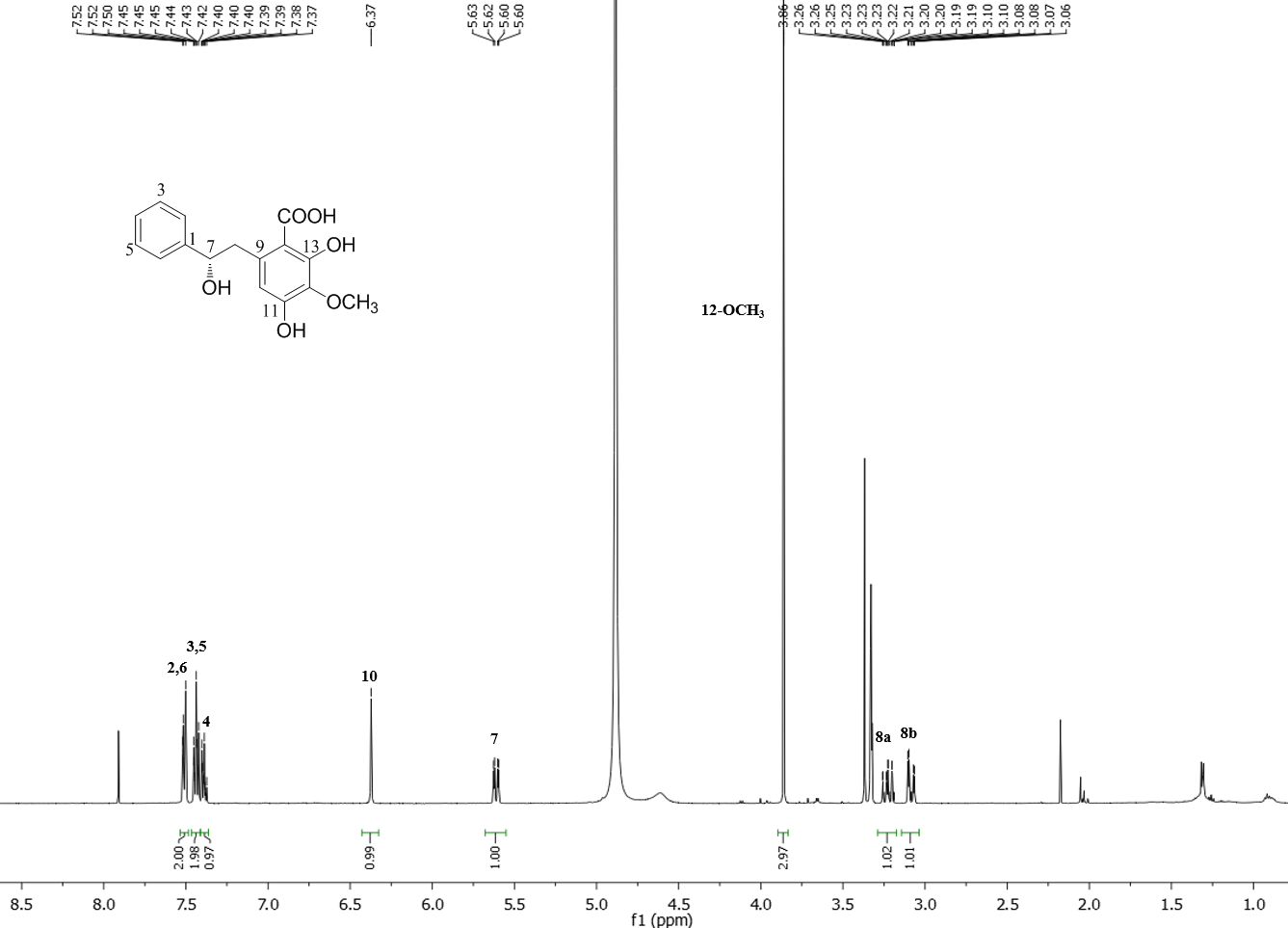


**Figure S1**. ^1^H NMR spectrum of compound **1**


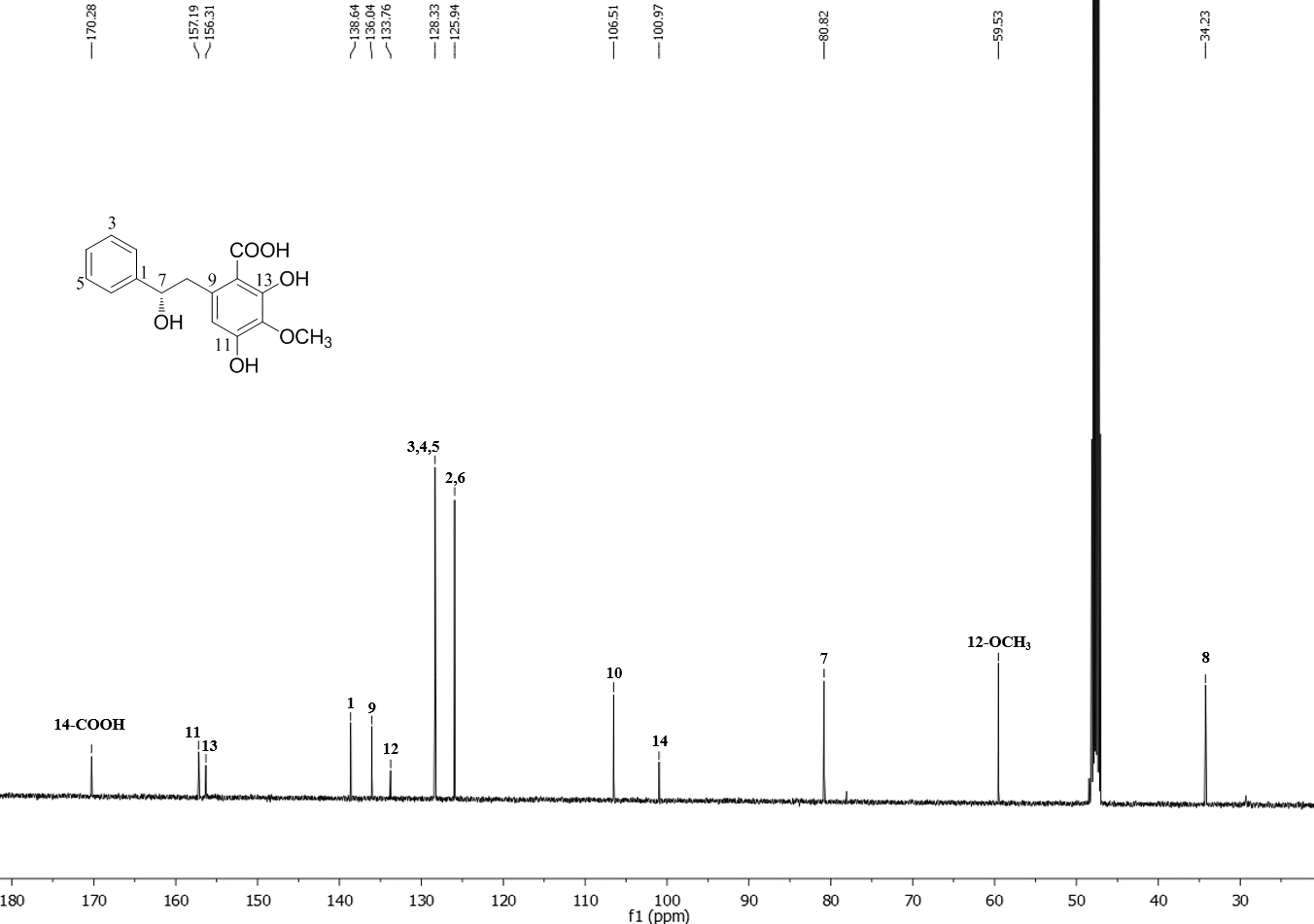


**Figure S2**. ^13^C NMR spectrum of compound **1**


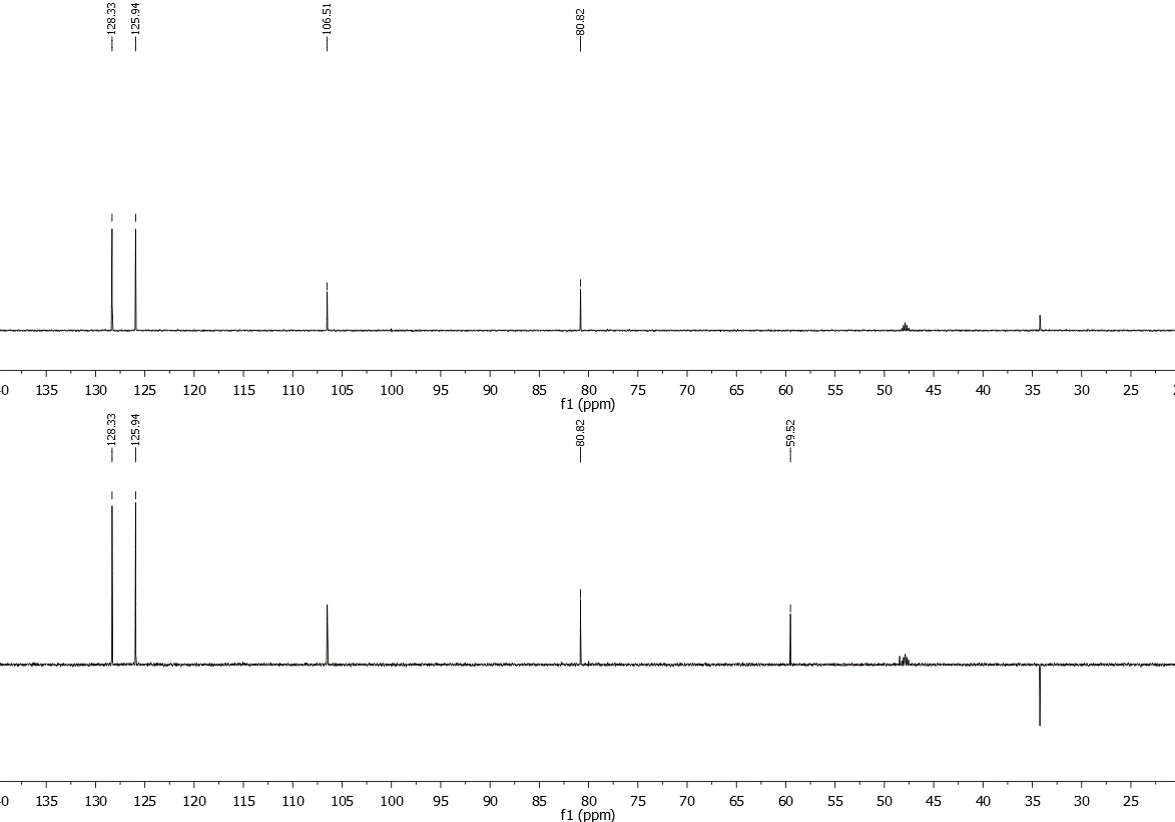


**Figure S3**. DEPT spectrum of compound **1**


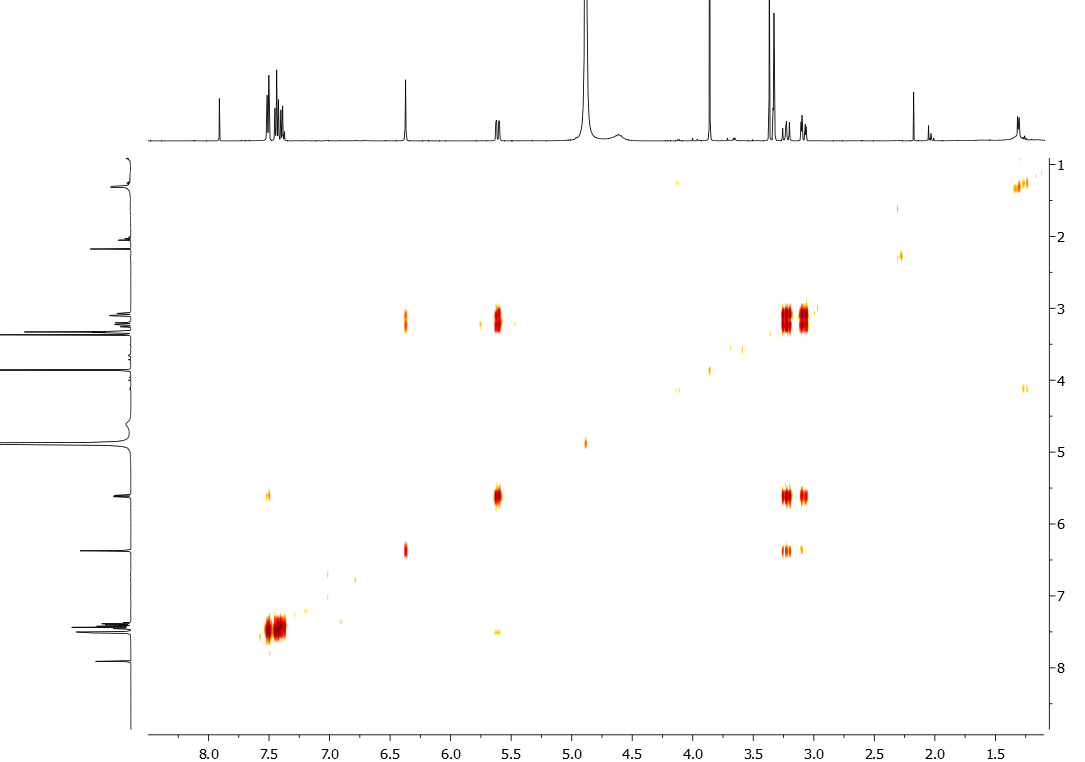


**Figure S4**. COSY spectrum of compound **1**


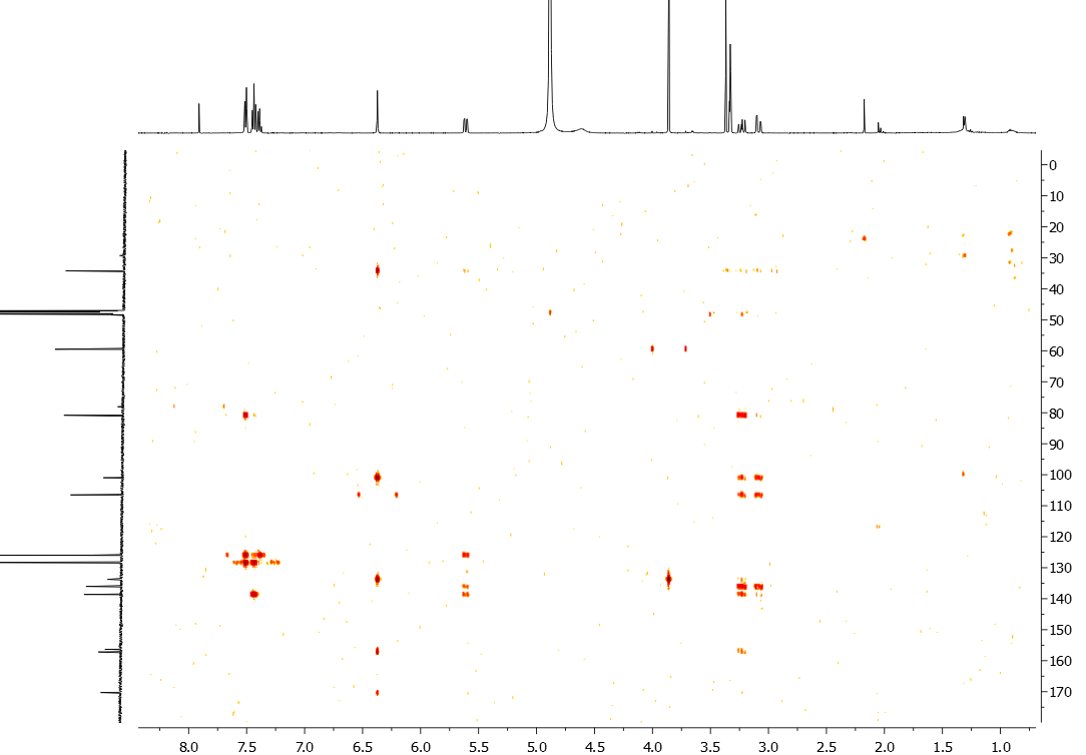


**Figure S5**. HMBC spectrum of compound **1**


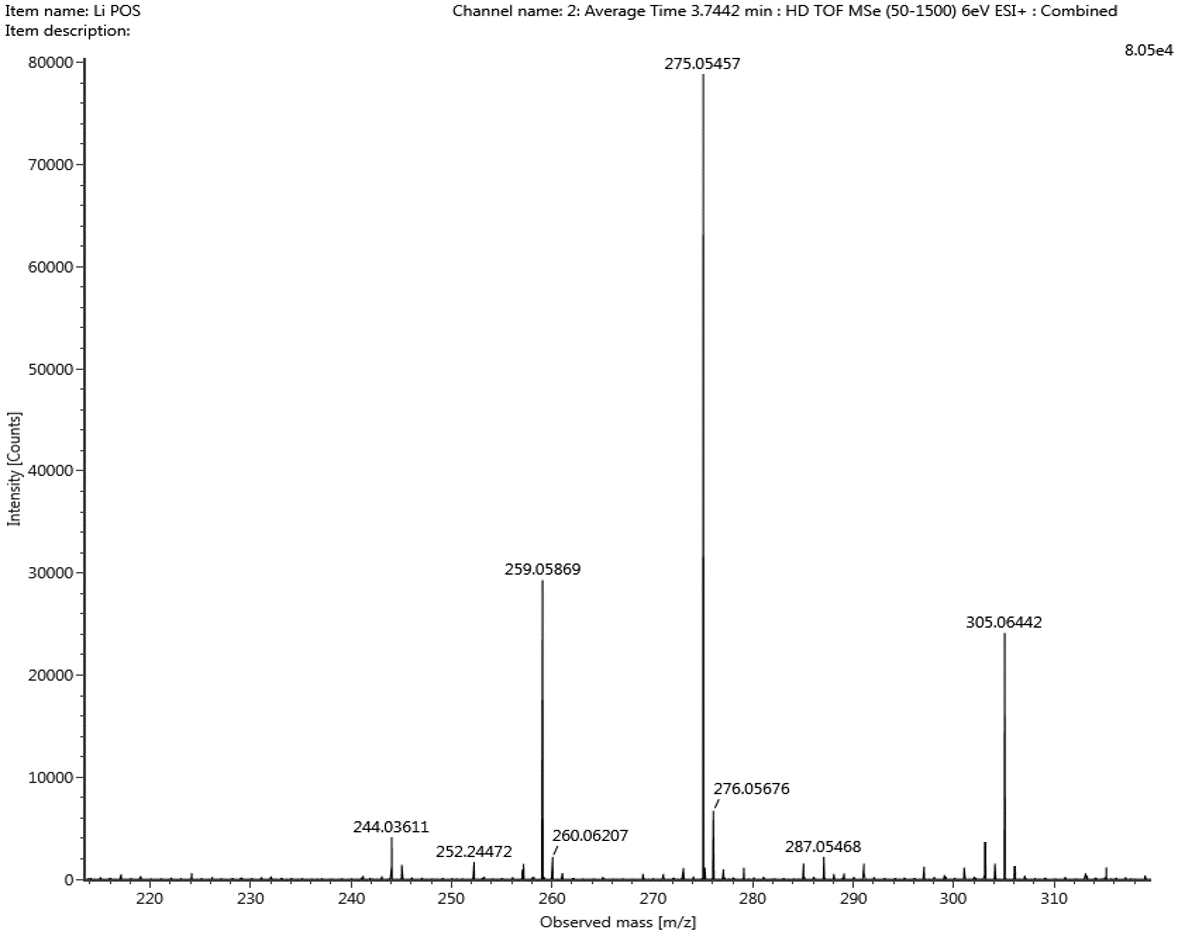


**Figure S6**. HRESIMS spectrum of compound **1**

**
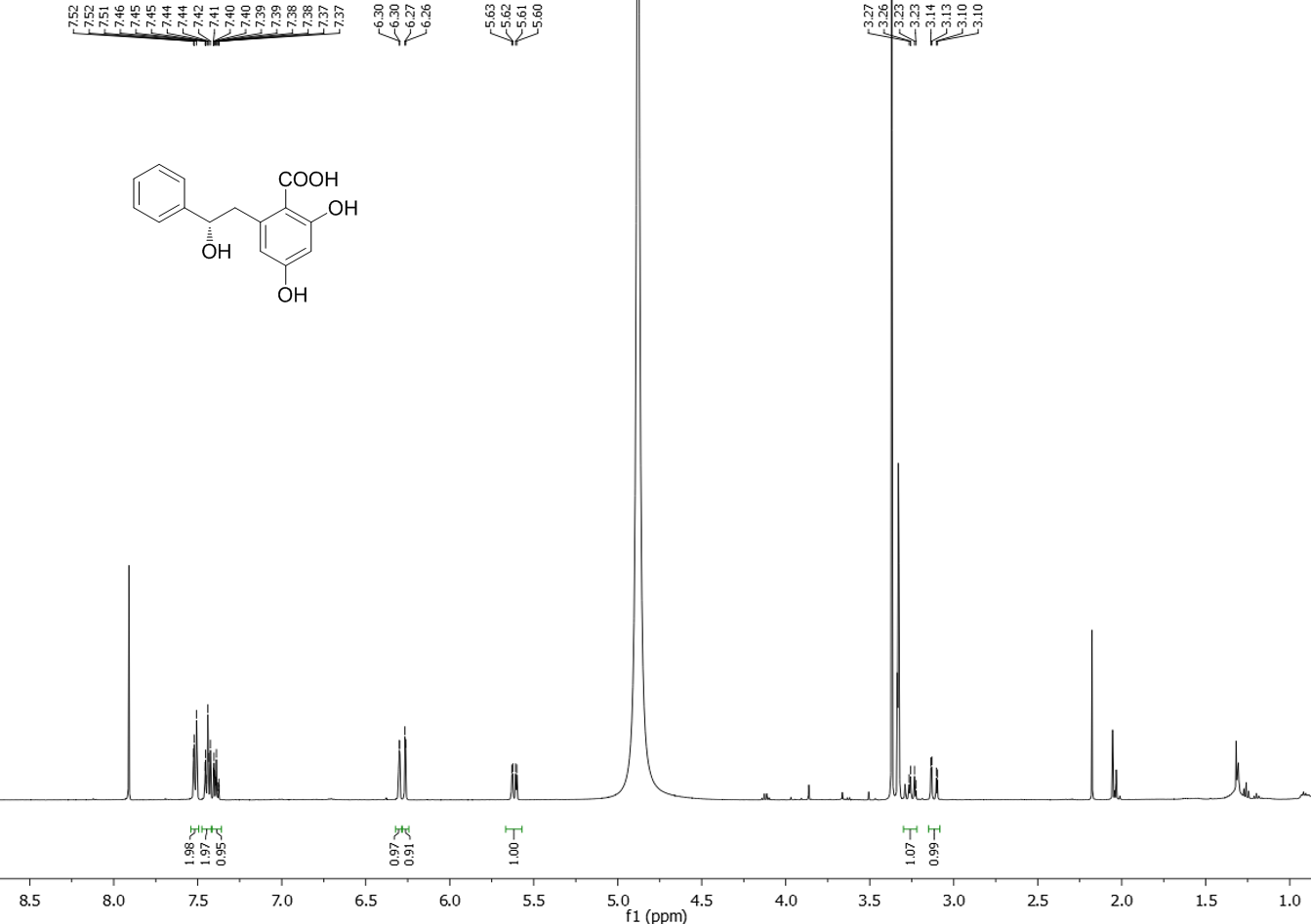
**

**Figure S7**. ^1^H NMR spectrum of compound **2**


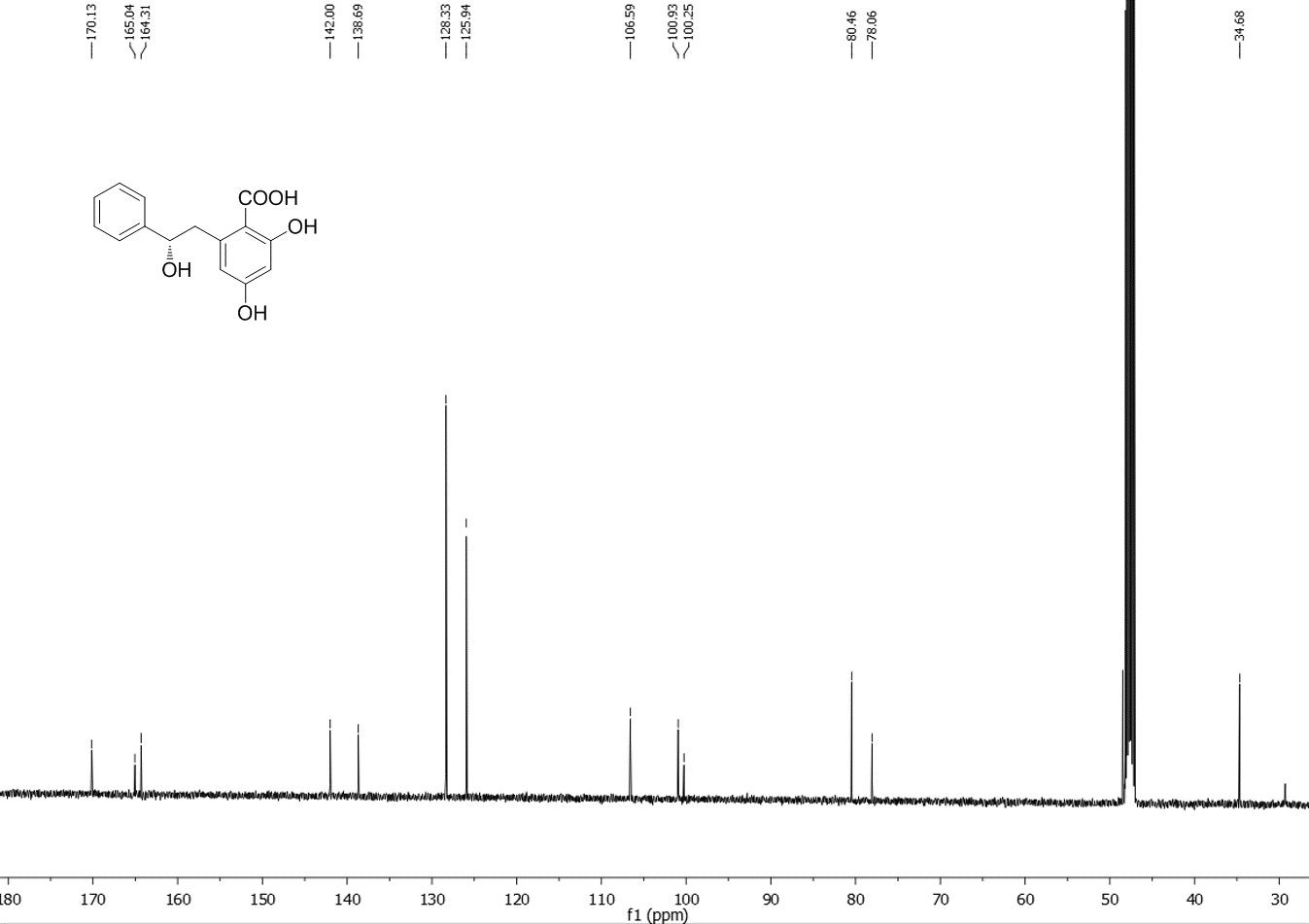


**Figure S8**. ^13^C NMR spectrum of compound **2**

**
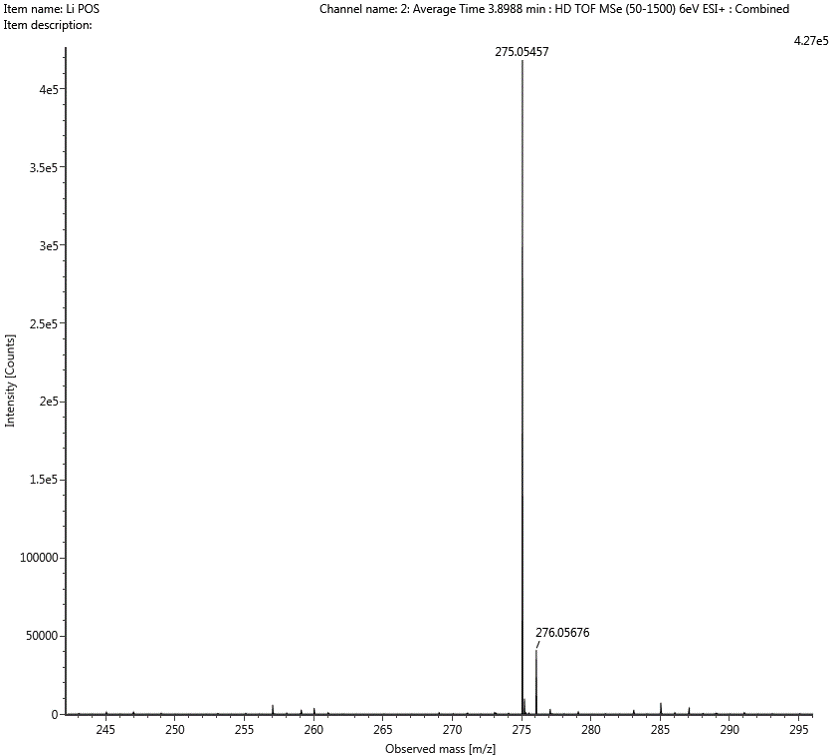
**

**Figure S9**. HRESIMS spectrum of compound **2**


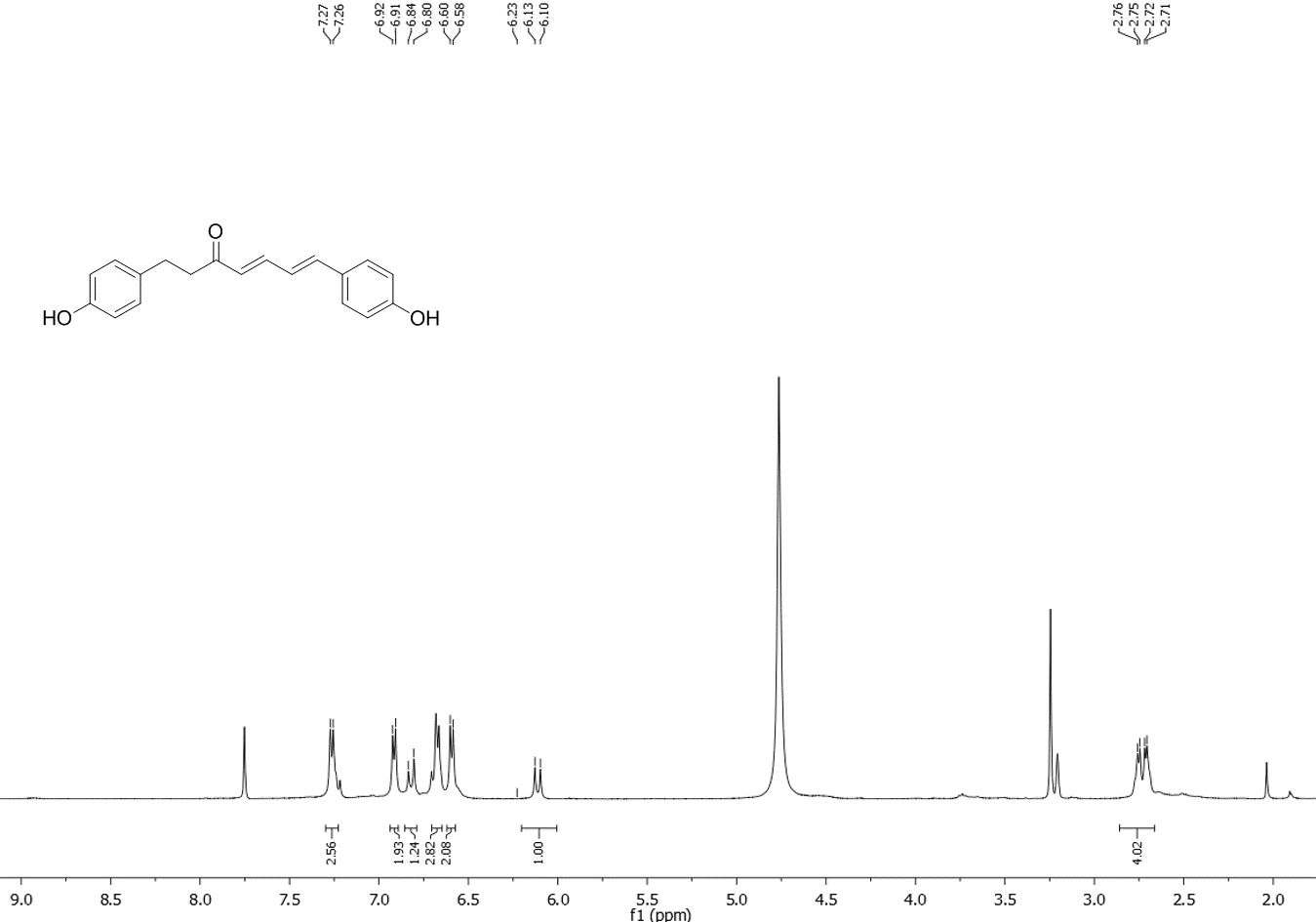


**Figure S10**. ^1^H NMR spectrum of compound **3**


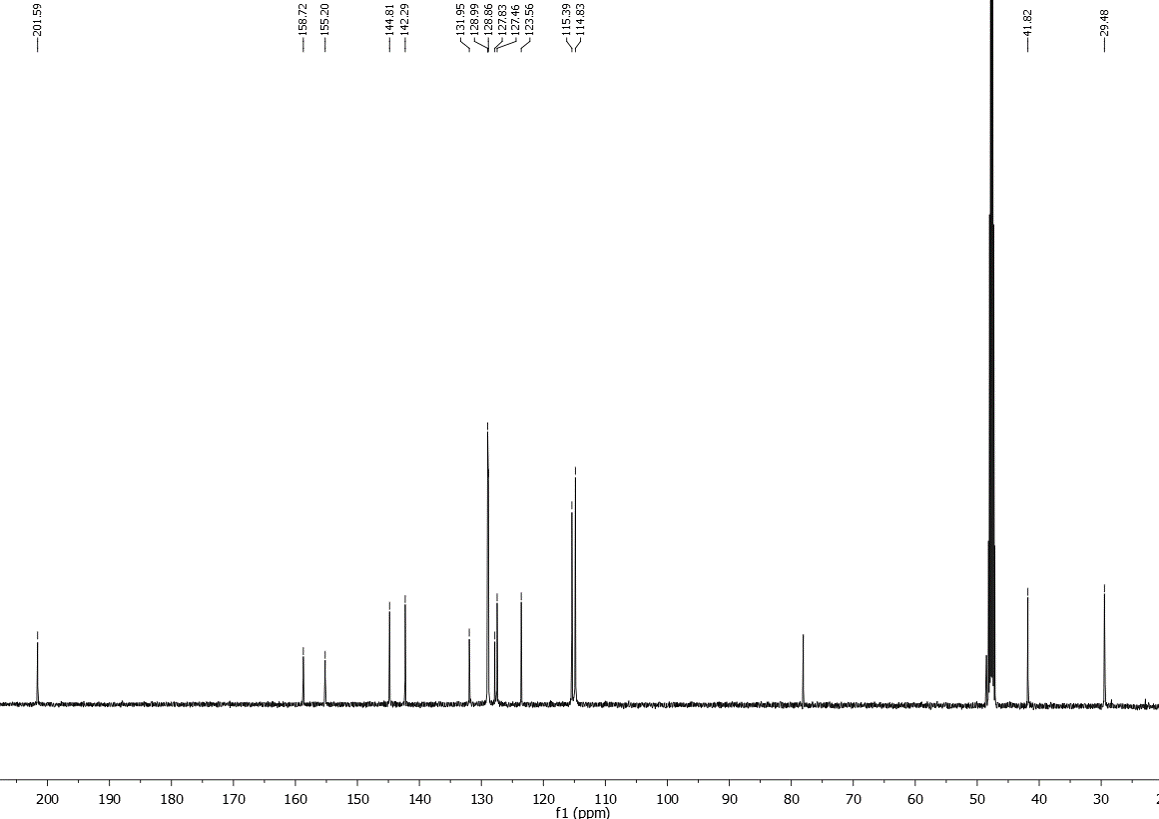


**Figure S11**. ^13^C NMR spectrum of compound **3**


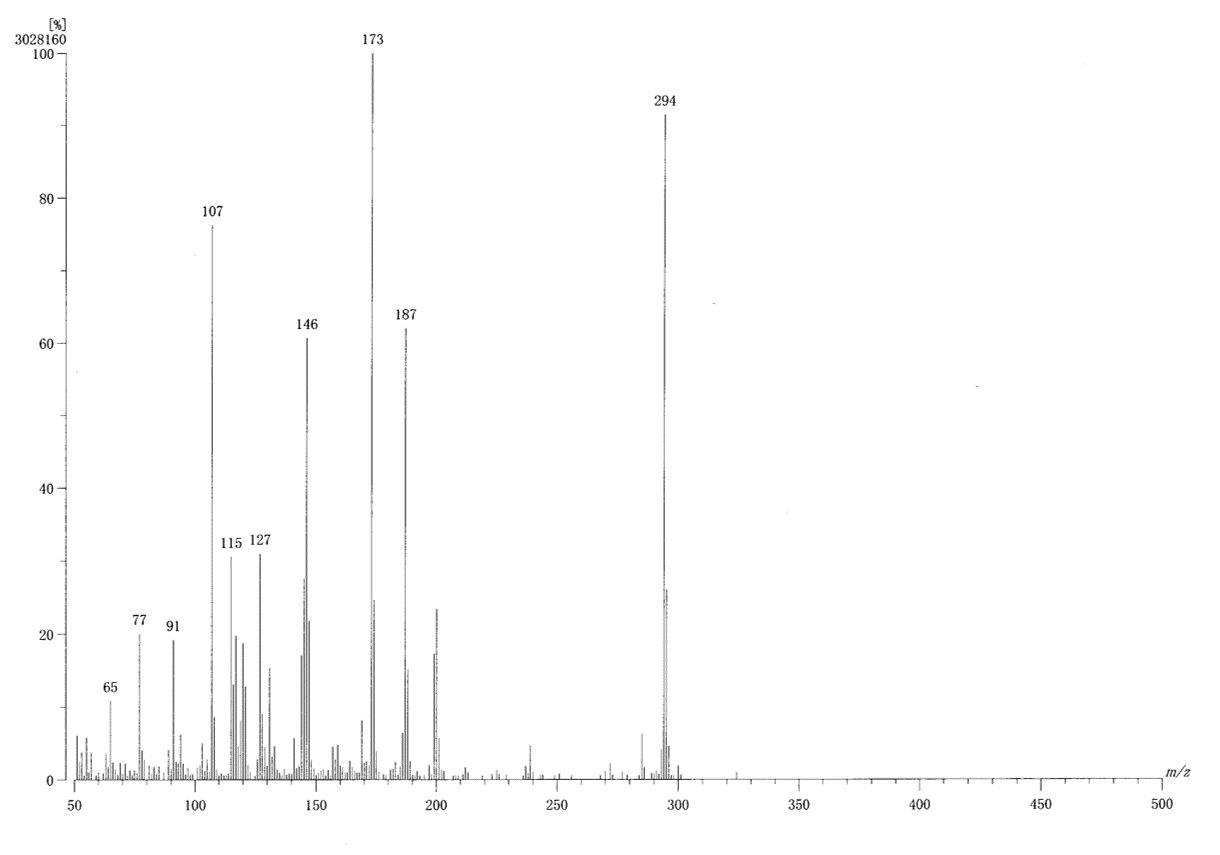


**Figure S12**. EIMS spectrum of compound **3**

**
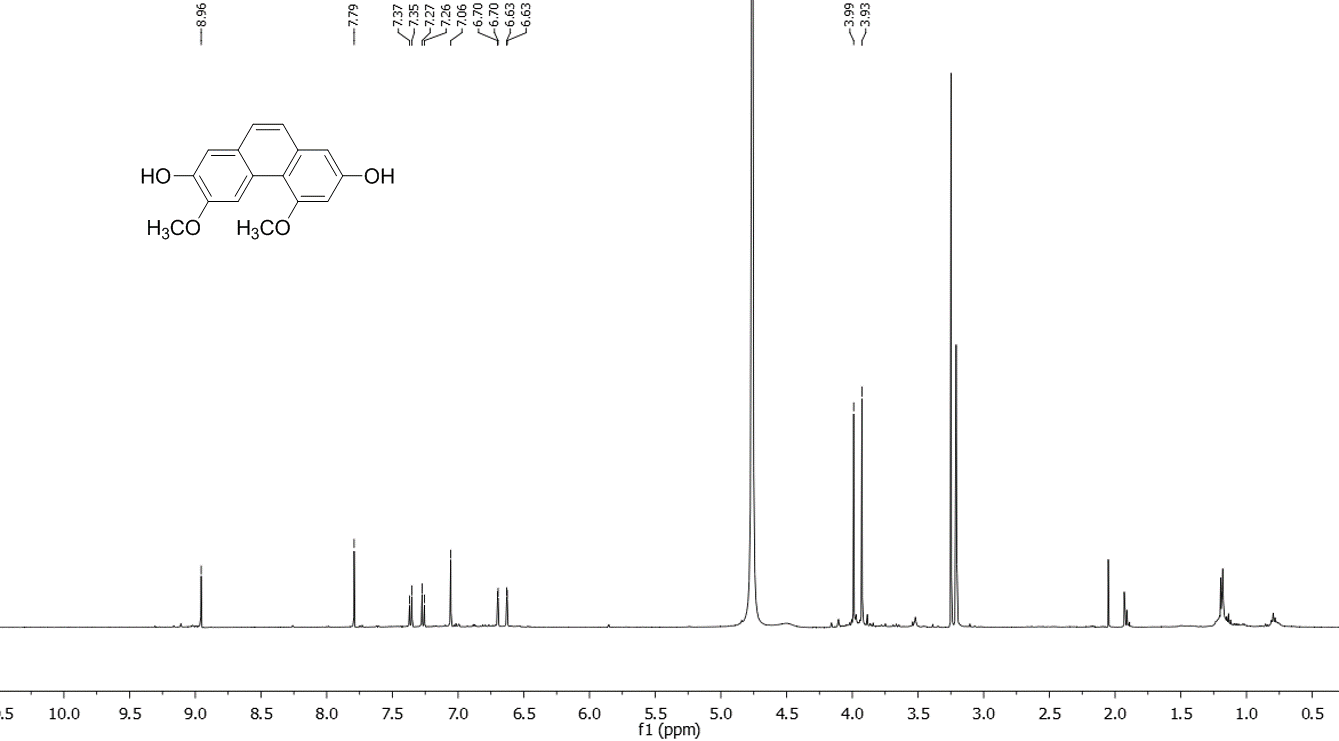
**

**Figure S13**. ^1^H NMR spectrum of compound **4**

**
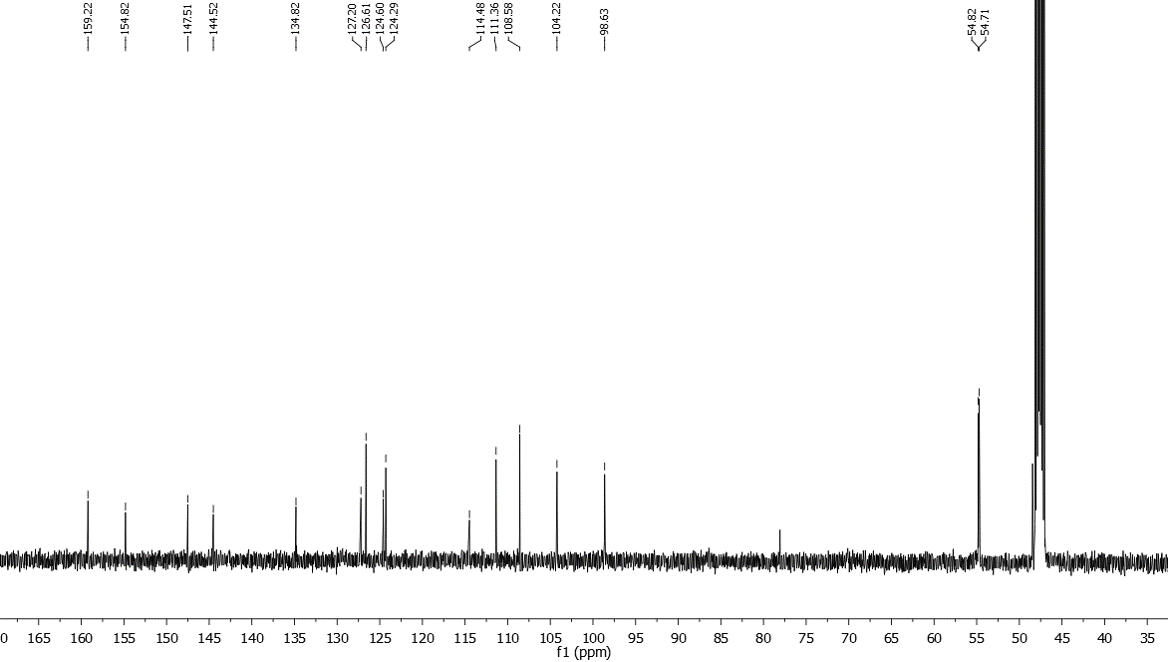
**

**Figure S14**. ^13^C NMR spectrum of compound **4**

**
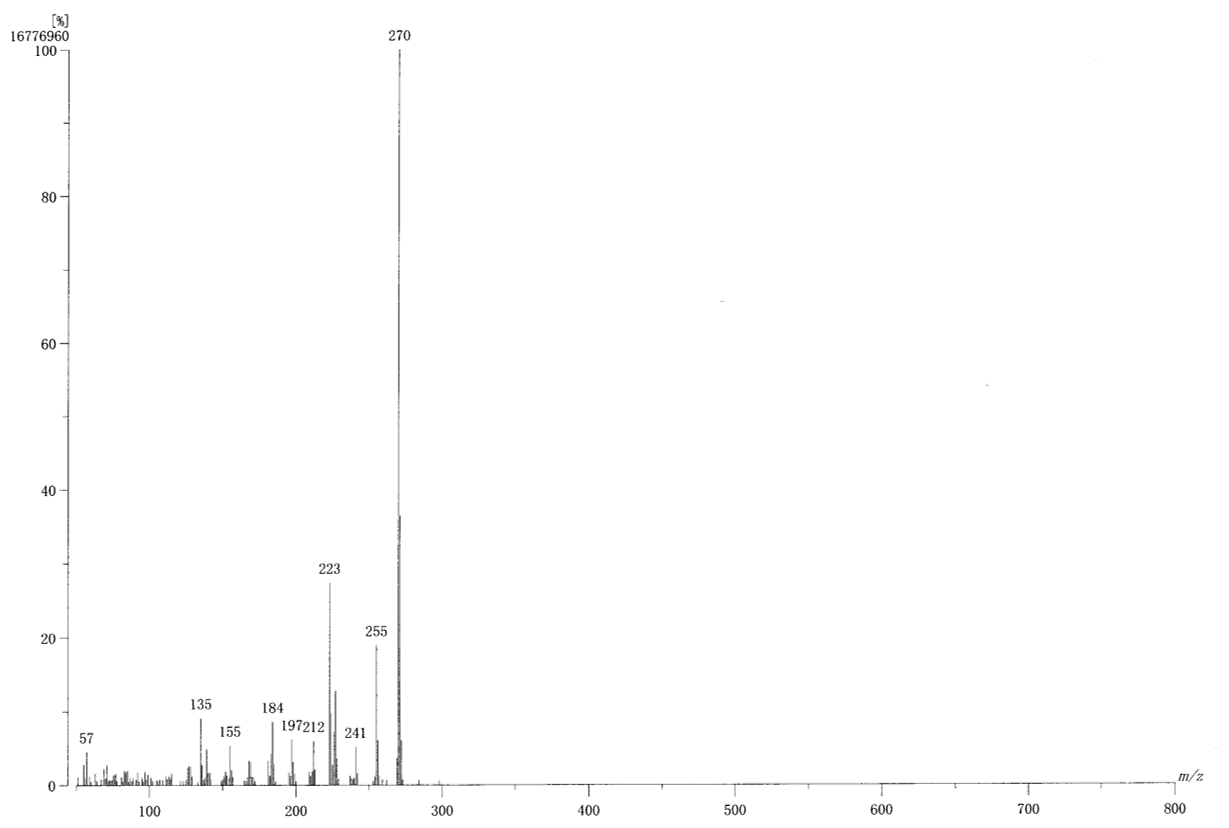
**

**Figure S15**. EIMS spectrum of compound **4**

**
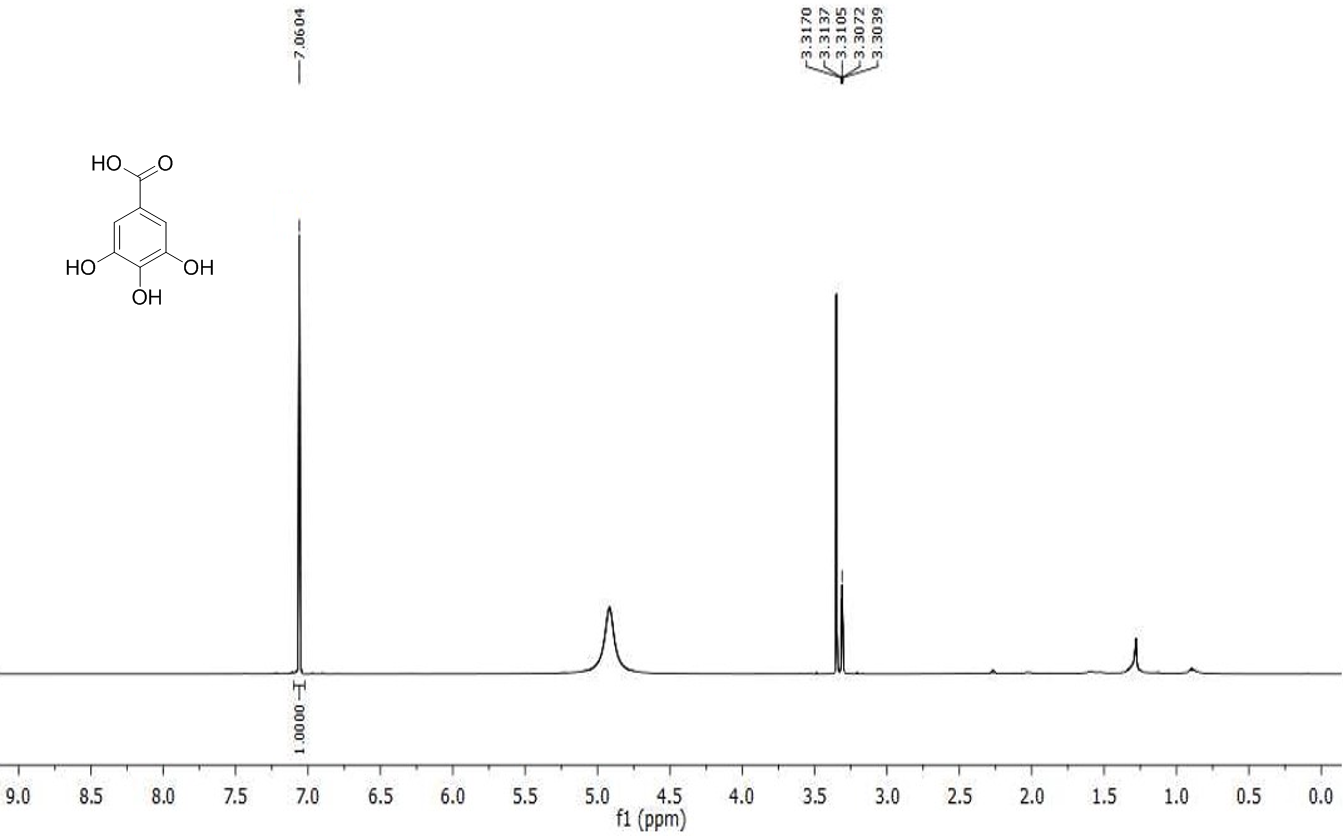
**

**Figure S16**. ^1^H NMR spectrum of compound **5**

**
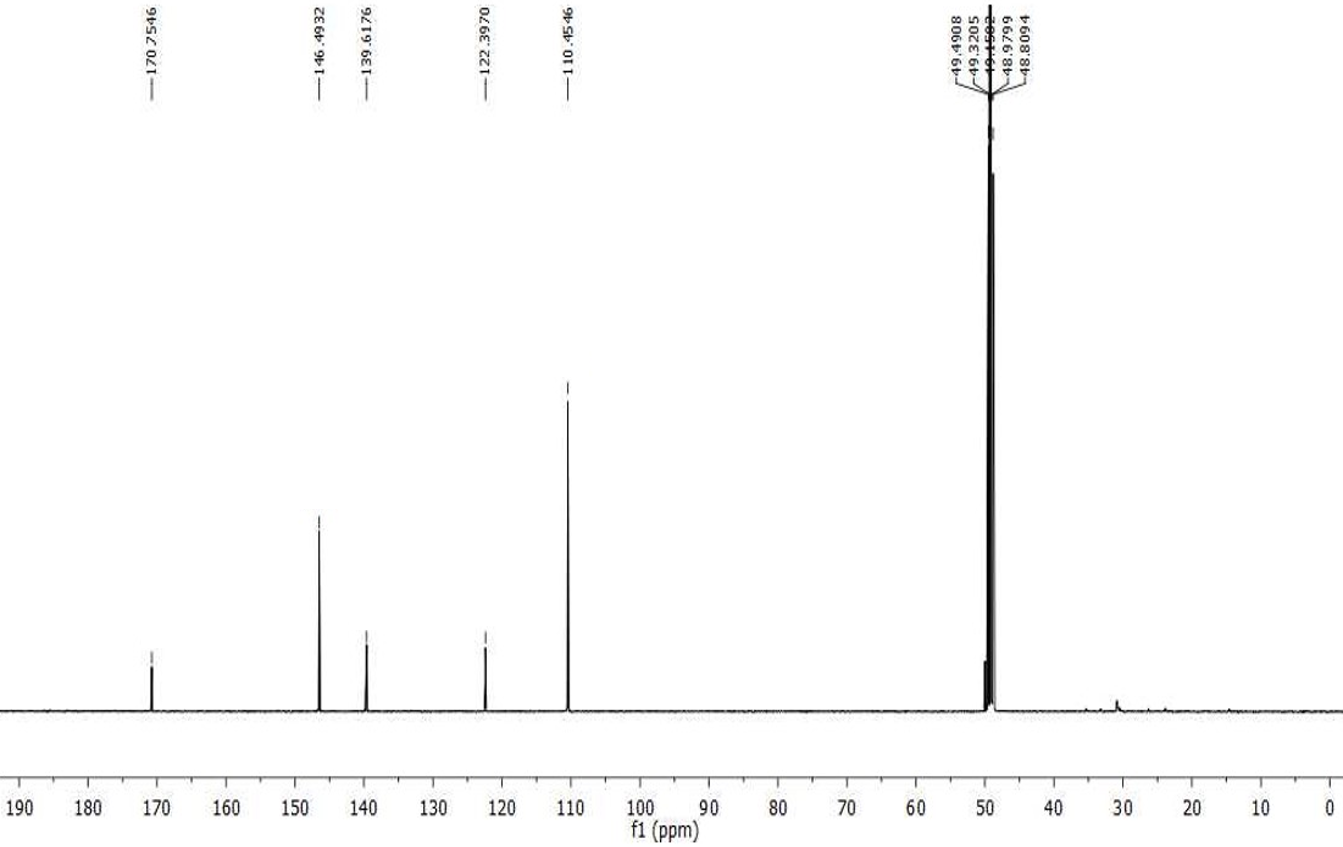
**

**Figure S17**. ^13^C NMR spectrum of compound **5**

**
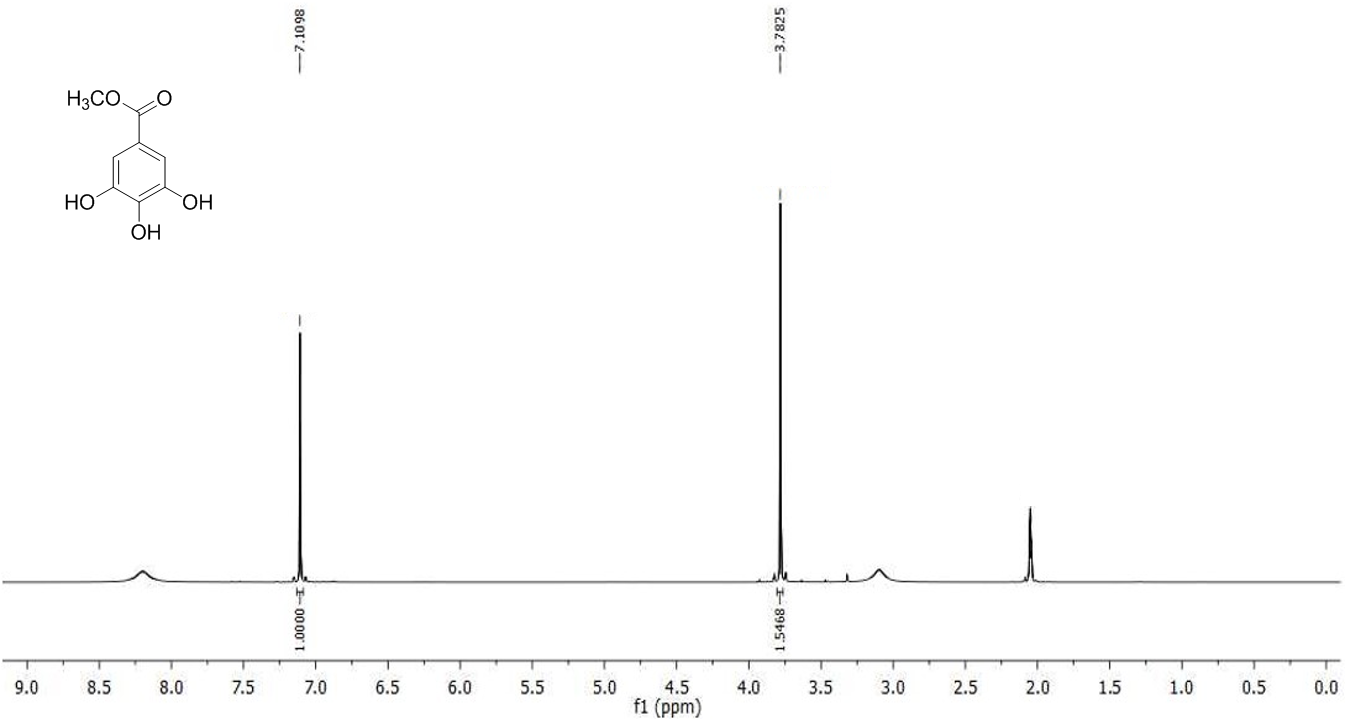
**

**Figure S18**. ^1^H NMR spectrum of compound **6**

**
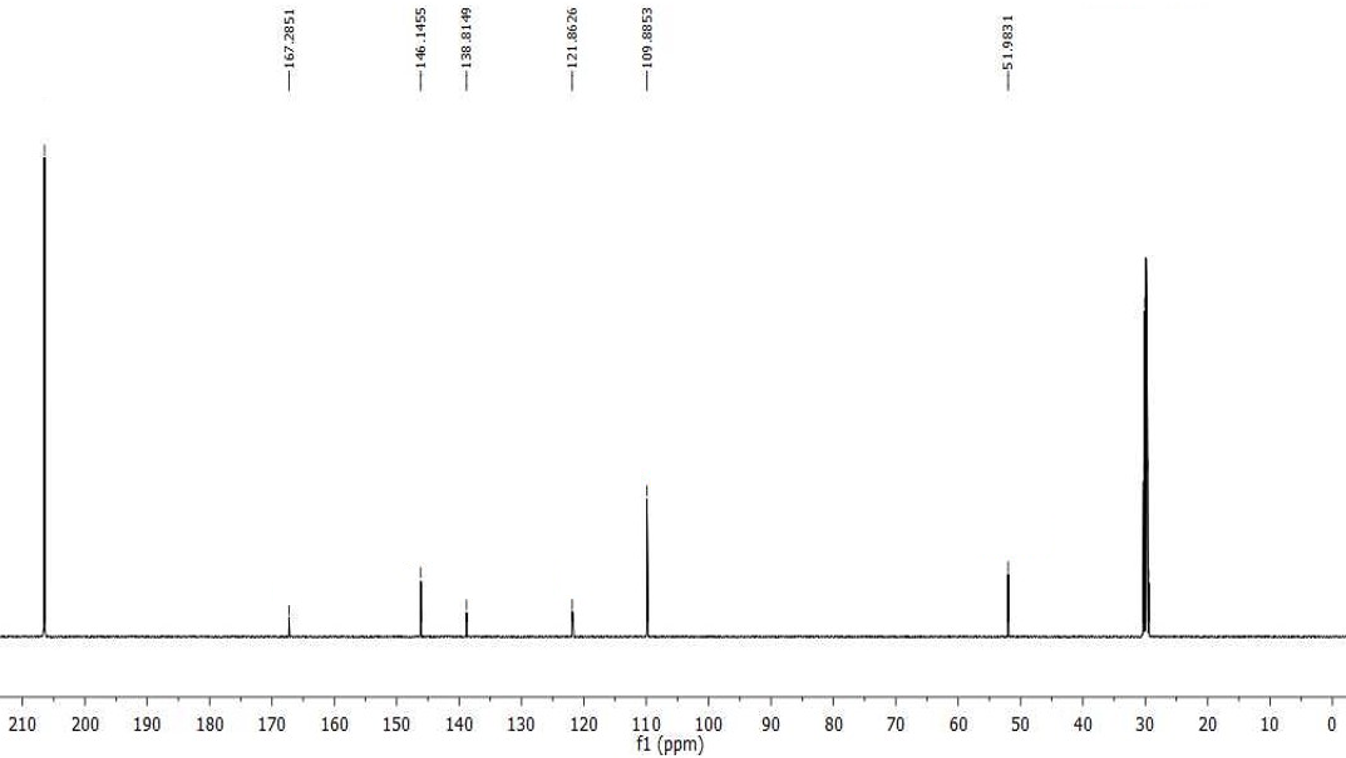
**

**Figure S19**. ^13^C NMR spectrum of compound **6**

**
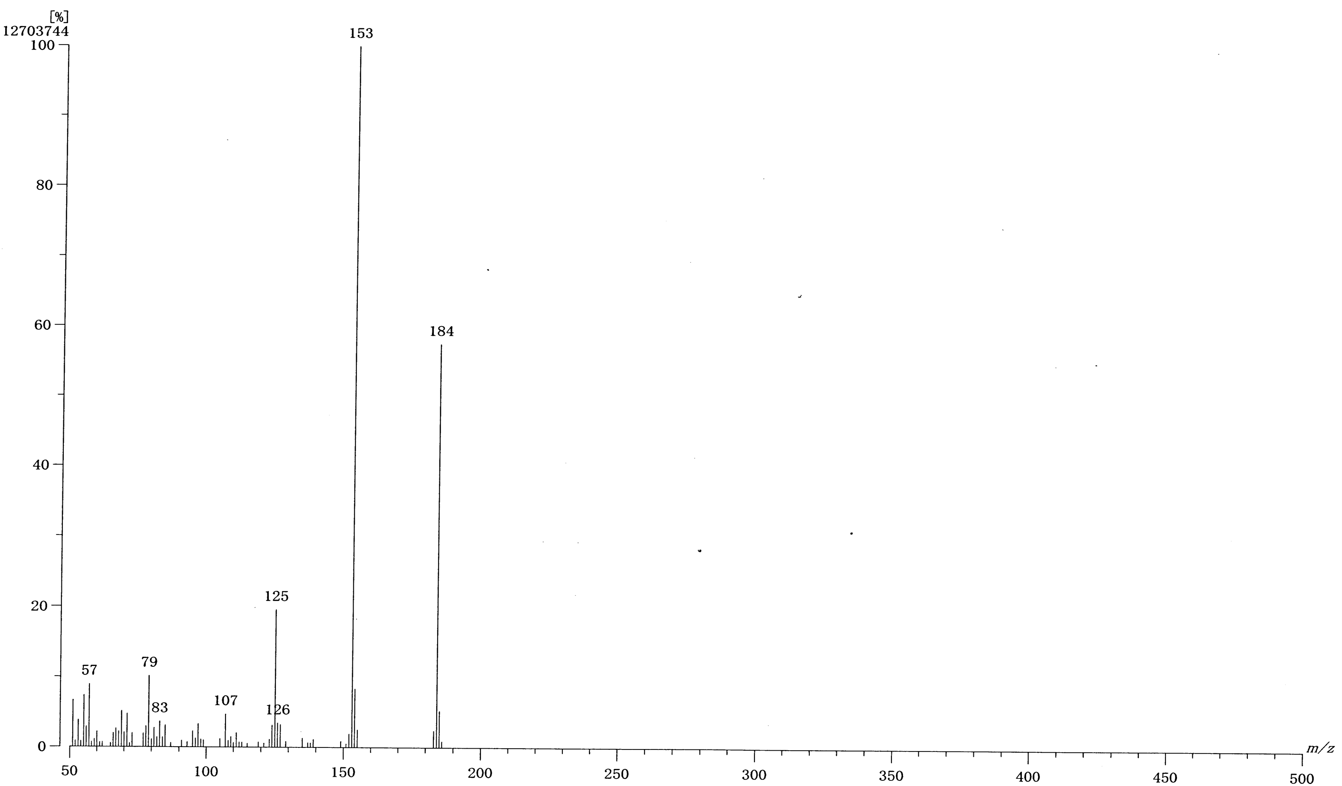
**

**Figure S20**. EIMS spectrum of compound **6**

**
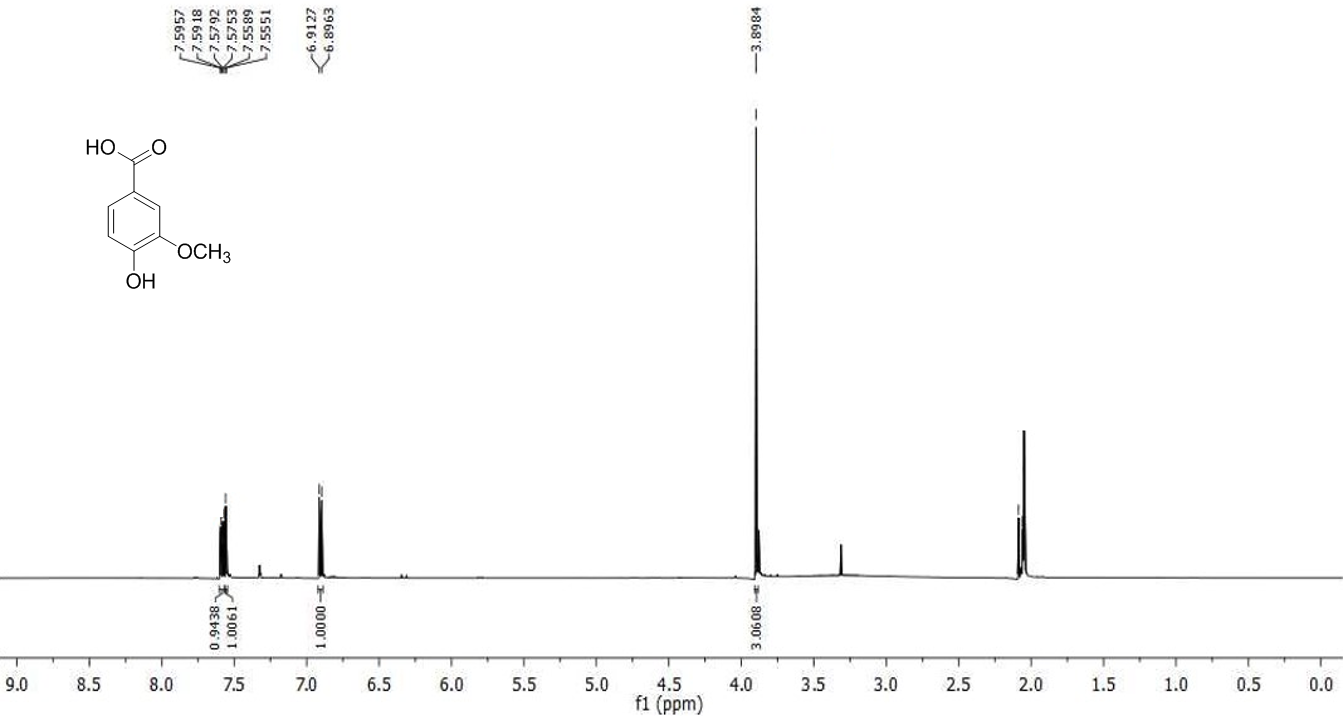
**

**Figure S21**. ^1^H NMR spectrum of compound **7**

**
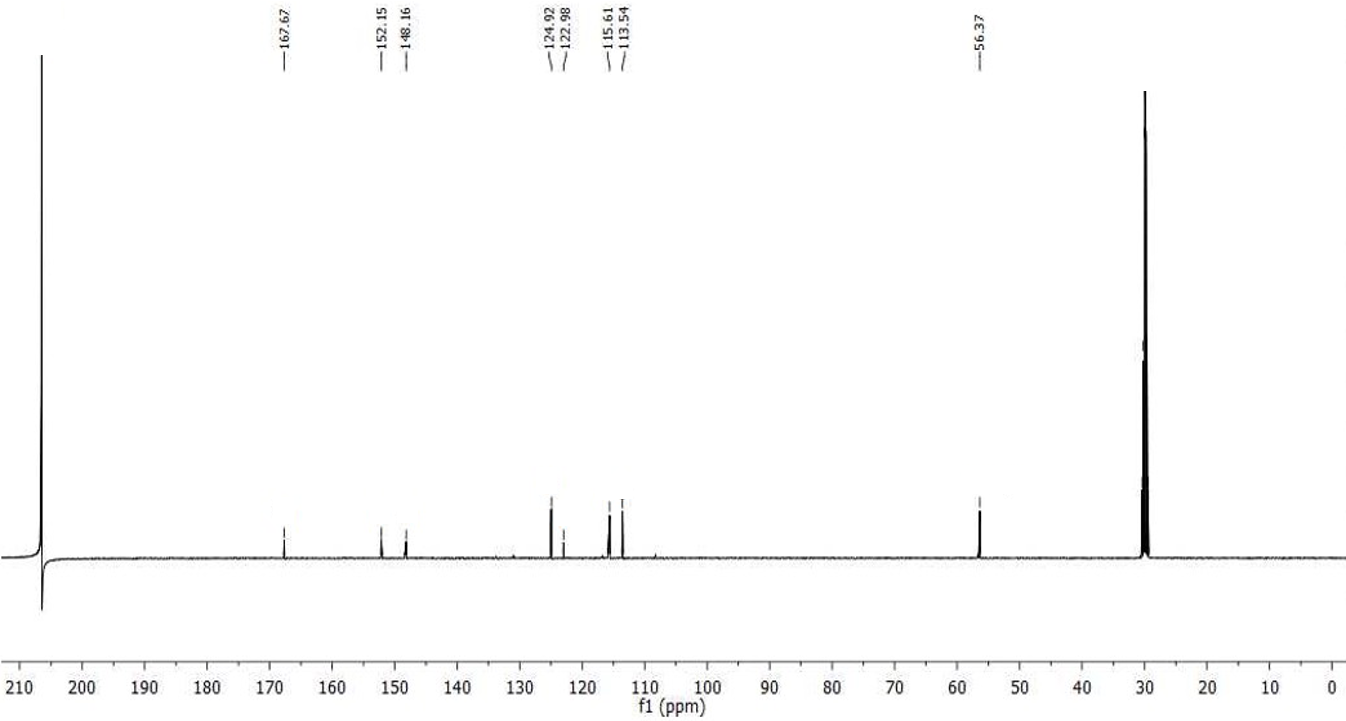
**

**Figure S22**. ^13^C NMR spectrum of compound **7**

**
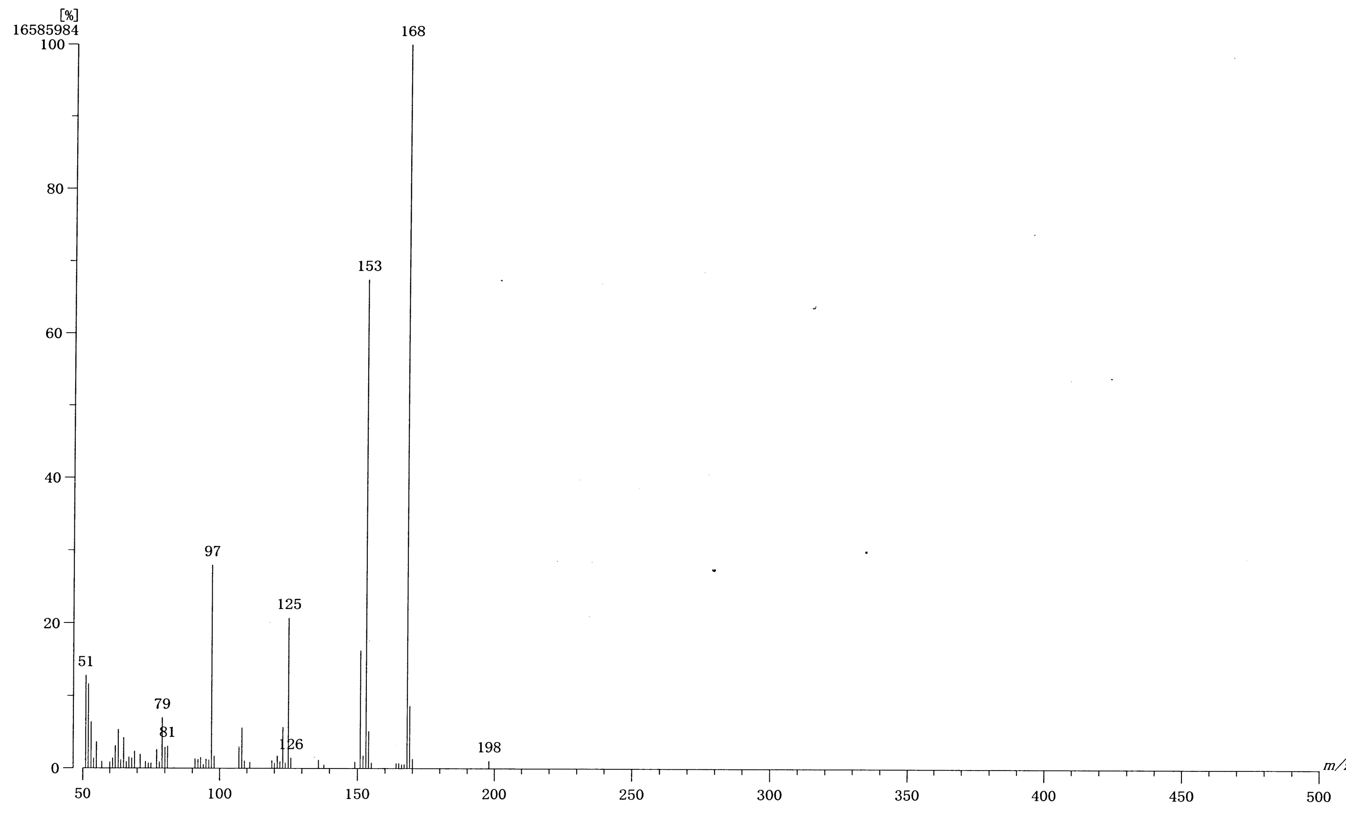
**

**Figure S23**. EIMS spectrum of compound **7**

**
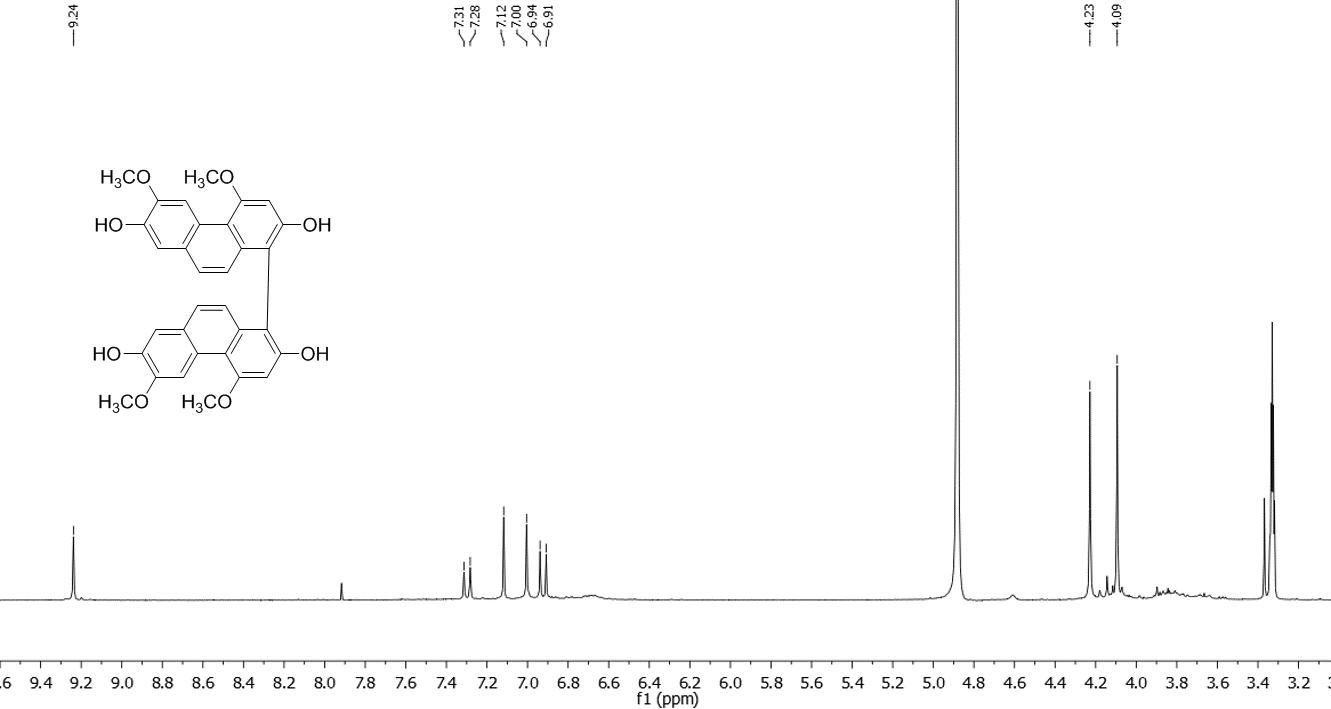
**

**Figure S24**. ^1^H NMR spectrum of compound **8**


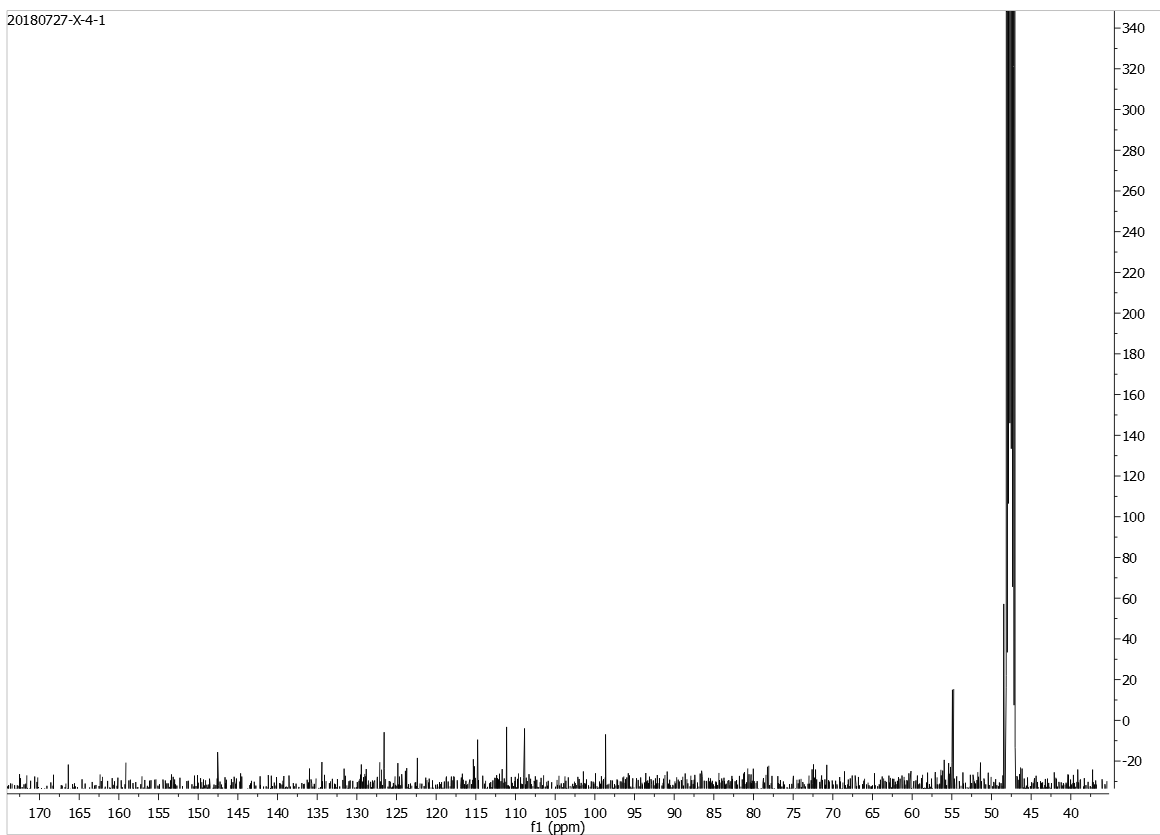


**Figure S25**. ^13^C NMR spectrum of compound **8**

**
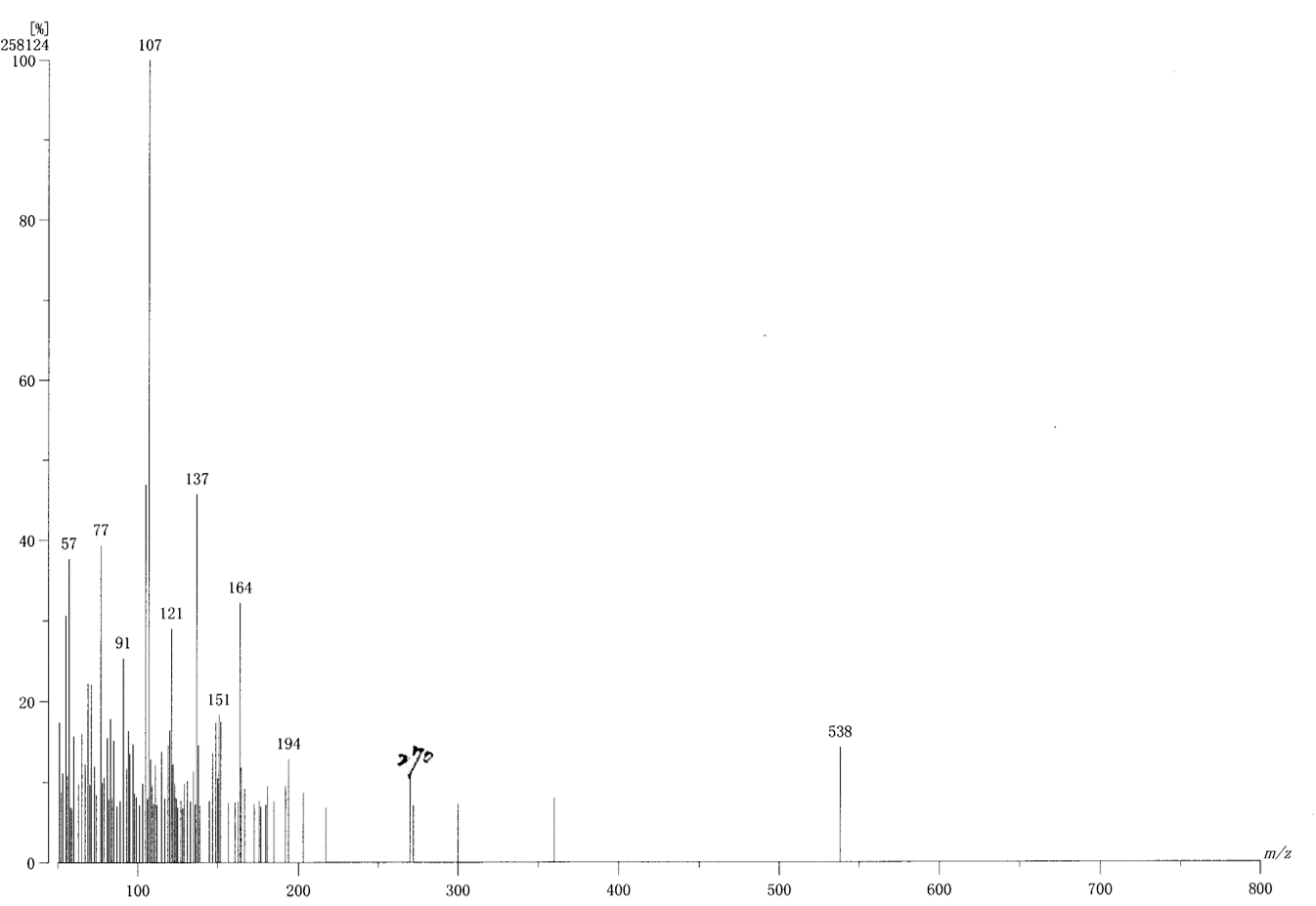
**

**Figure S26**. EIMS spectrum of compound **8**

**
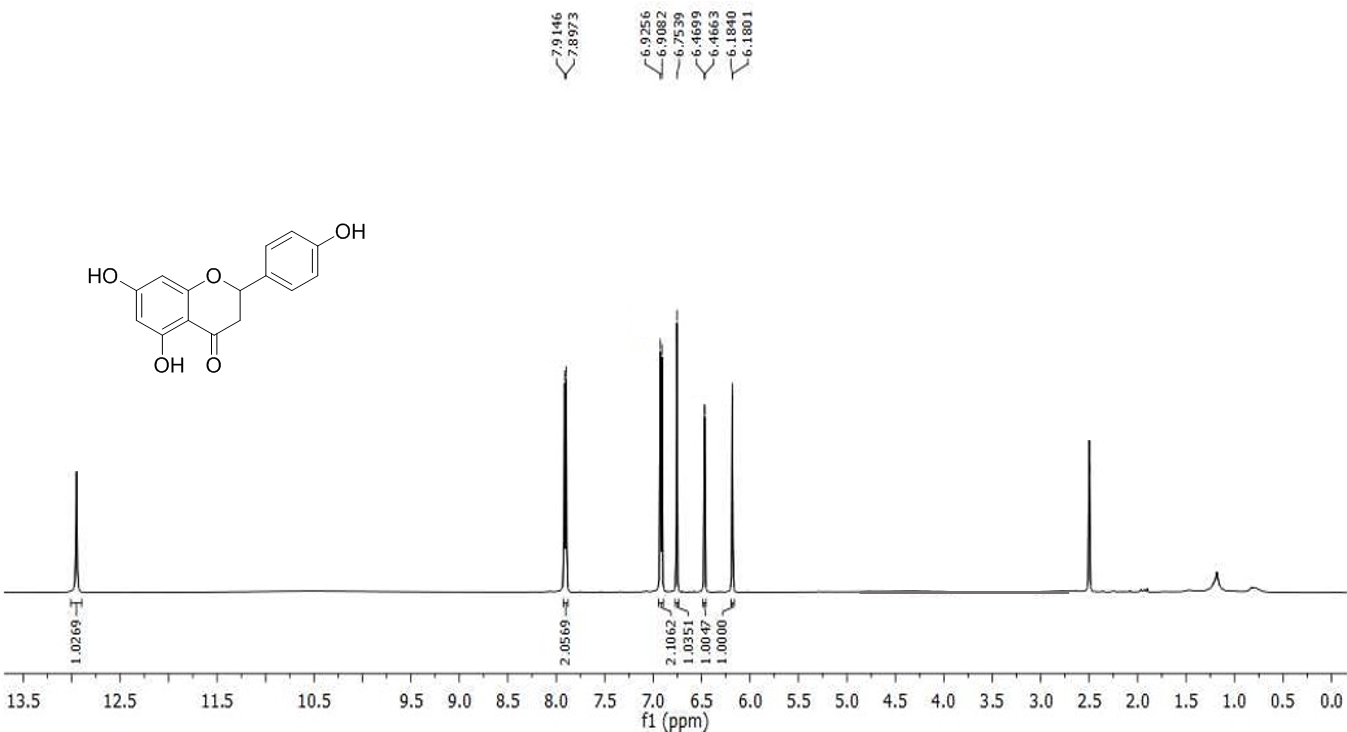
**

**Figure S27**. ^1^H NMR spectrum of compound **9**

**
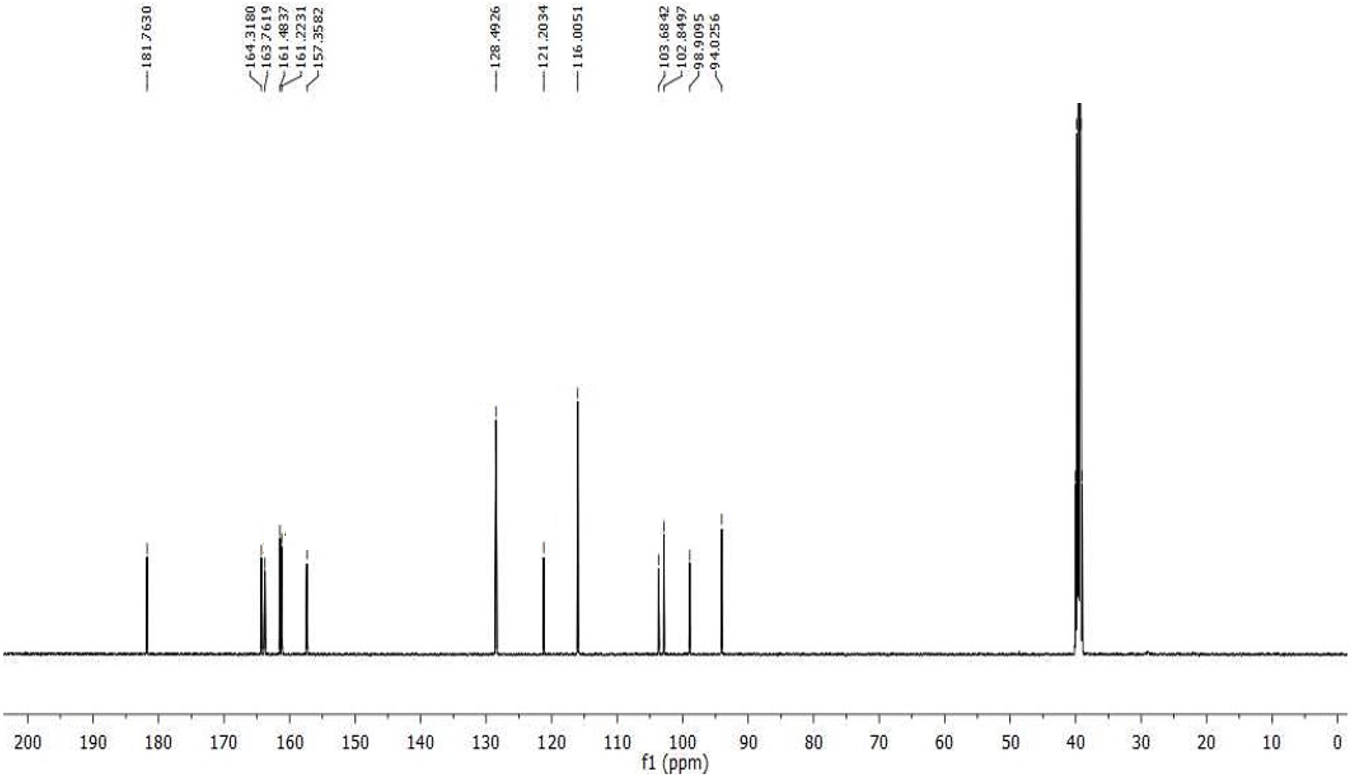
**

**Figure S28**. ^13^C NMR spectrum of compound **9**

**
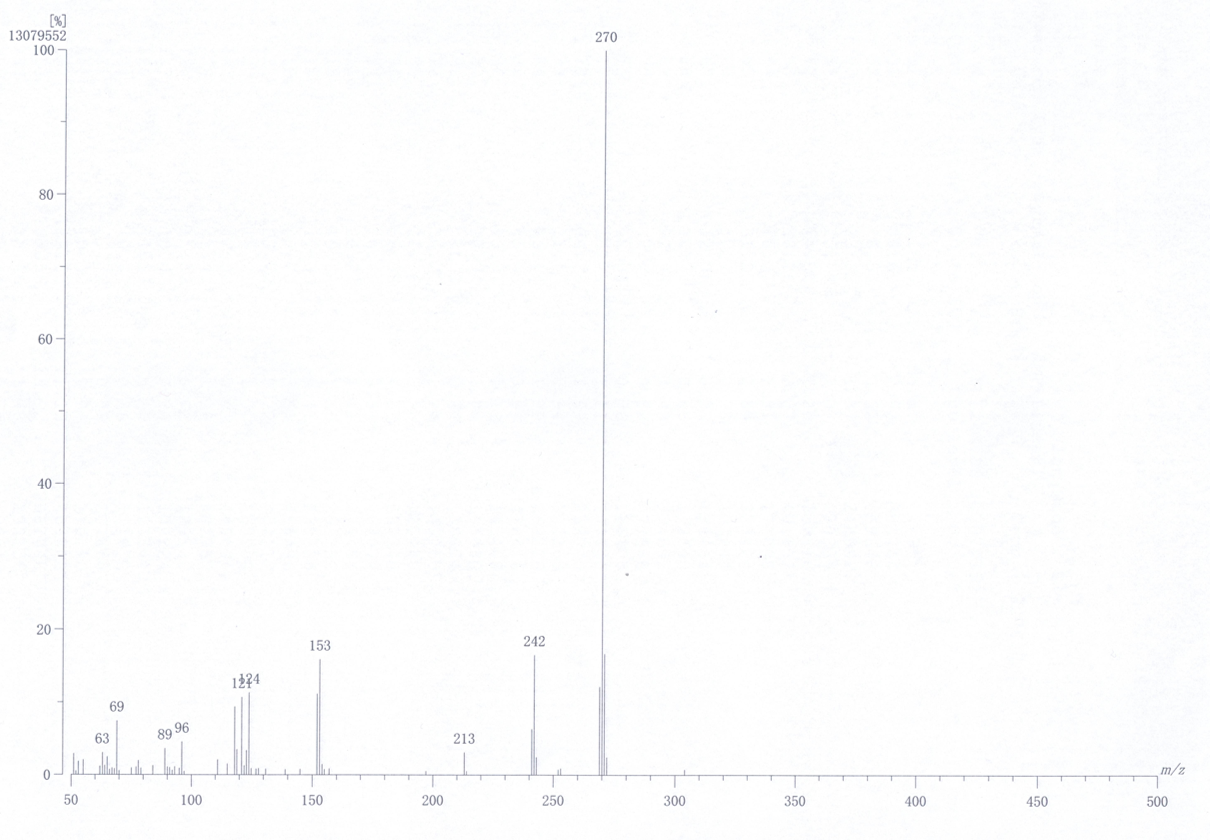
**

**Figure S29**. EIMS spectrum of compound **9**
